# Vertical inhibition of p110α/AKT and N‐cadherin enhances treatment efficacy in *PIK3CA*‐aberrated ovarian cancer cells

**DOI:** 10.1002/1878-0261.13761

**Published:** 2024-11-14

**Authors:** Shibo Zhang, Hei Ip Hong, Victor C. Y. Mak, Yuan Zhou, Yiling Lu, Guanglei Zhuang, Lydia W. T. Cheung

**Affiliations:** ^1^ School of Biomedical Sciences, Li Ka Shing Faculty of Medicine The University of Hong Kong China; ^2^ Sun Yat‐sen University Cancer Center, State Key Laboratory of Oncology in South China, Collaborative Innovation Center for Cancer Medicine, Guangdong Key Laboratory of Nasopharyngeal Carcinoma Diagnosis and Therapy Guangzhou China; ^3^ Division of Cancer Medicine, Department of Genomic Medicine UT MD Anderson Cancer Centre Houston TX USA; ^4^ State Key Laboratory of Systems Medicine for Cancer, Department of Obstetrics and Gynecology, Ren Ji Hospital, Shanghai Cancer Institute Shanghai Jiao Tong University School of Medicine China; ^5^ Shanghai Key Laboratory of Gynecologic Oncology, Ren Ji Hospital Shanghai Jiao Tong University School of Medicine China

**Keywords:** cytoplasmic YAP, N‐cadherin, p110α, PI3K, *PIK3CA*, serous ovarian cancer

## Abstract

Phosphatidylinositol‐4,5‐bisphosphate 3‐kinase catalytic subunit alpha [*PIK3CA*, encoding PI3Kalpha (also known as p110α)] is one of the most commonly aberrated genes in human cancers. In serous ovarian cancer, *PIK3CA* amplification is highly frequent but *PIK3CA* point mutation is rare. However, whether *PIK3CA* amplification and *PIK3CA* driver mutations have the same functional impact in the disease is unclear. Here, we report that both *PIK3CA* amplification and E545K mutation are tumorigenic. While the protein kinase B (AKT) signaling axis was activated in both E545K knock‐in cells and *PIK3CA*‐overexpressing cells, the mitogen‐activated protein kinase 3/1 (ERK1/2) pathway was induced selectively by E545K mutation but not *PIK3CA* amplification. Intriguingly, AKT signaling in these *PIK3CA*‐aberrated cells increased transcriptional coactivator YAP1 (YAP) Ser127 phosphorylation and thereby cytoplasmic YAP levels, which in turn increased cell migration through Ras‐related C3 botulinum toxin substrate 1 (RAC1) activation. In addition to the altered YAP signaling, AKT upregulated N‐cadherin expression, which also contributed to cell migration. Pharmacological inhibition of N‐cadherin reduced cell migratory potential. Importantly, co‐targeting N‐cadherin and p110α/AKT caused additive reduction in cell migration *in vitro* and metastases formation *in vivo*. Together, this study reveals the molecular pathways driven by the *PIK3CA* aberrations and the exploitable vulnerabilities in *PIK3CA*‐aberrated serous ovarian cancer cells.

## Graphical abstract

*PIK3CA* amplification and *PIK3CA* mutation enhance ovarian tumorigenicity through an activation of AKT, which phosphorylates YAP at Ser127. This Ser127 phosphorylation leads to the retention of YAP in the cytoplasm and triggers RAC1 activity, promoting cell migration. Additionally, AKT activation increases expression of N‐cadherin, which further enhances cell migration. This p110α/AKT/N‐cadherin signaling axis represents a potential therapeutic vulnerability.

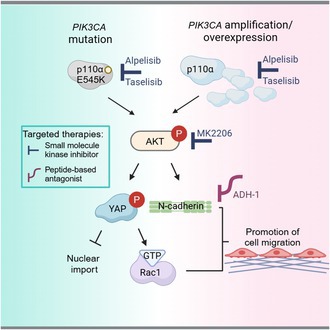

**Abbreviations**
AKT: protein kinase B
CI: confidence interval
DEGs: differentially expressed genes
DEPs: differentially expressed proteins
GO: gene ontology
GSEA: gene set enrichment analysis
HDR: homology directed repair
HNSCC: head and neck squamous carcinoma
HR: hazard ratio
HRP: horseradish peroxidase
IHC: immunohistochemistry
IRS: immunoreactive score
KEGG: Kyoto Encyclopedia of Genes and Genomes
KM: Kaplan–Meier
MAPK: mitogen‐activated protein kinase
NES: normalized enrichment score
PAM: protospacer adjacent motif
PI3K: phosphoinositide‐3 kinase
RAC1: Ras‐related C3 botulinum toxin substrate 1
RNA‐seq: RNA sequencing
RPPA: reverse phase protein array
TCGA: The Cancer Genome Atlas

## 1 Introduction

p110α (encoded by *PIK3CA* on chromosome 3q26.3) is a catalytic subunit of the class IA phosphoinositide‐3 kinase (PI3K). The primary reaction driven by p110α is the conversion of PtdIns (3,4,5)‐trisphosphate (PIP3) from PtdIns (4,5)‐bisphosphate (PIP2), which is a lipid second messenger to initiate signaling for cellular functions and disease development including cancers [1]. p110α contains five domains: adaptor‐binding domain, Ras‐binding domain, C2 domain, helical domain, and kinase domain [2]. p110α forms obligate heterodimer with the p85 regulatory subunit through its helical domain [1, 2]. p85 stabilizes p110α by preventing p110α from degradation. p85 also inhibits p110α kinase activity but this inhibition is relieved by activated receptor tyrosine kinases.

Point mutations in *PIK3CA*, with the hotspots E545K and H1047R, are among the 10 most frequent events in pan‐cancer samples [3, 4, 5]. E545K in the helical domain disrupts the inhibitory interaction between p110α and p85, whereas H1047R located at the kinase domain leads to constitutively active state of the protein [6, 7]. These hotspot mutants are well‐characterized functional drivers that cause pathway deregulation and promote tumorigenesis [2, 8, 9]. According to The Cancer Genome Atlas (TCGA), the cancer types with frequent *PIK3CA* mutations are that of endometrium (46%), breast (31%), and colon (25%). In ovarian cancer, the occurrence of *PIK3CA* mutations varies among histological subtypes (serous: 0.3–3%; clear cell: 23–50%, endometrioid and mucinous: up to 25%) as shown in TCGA and independent studies [10, 11, 12, 13]. It is noteworthy that while *PIK3CA* mutation is rare in the serous subtype, high prevalence of *PIK3CA* amplification is observed (TCGA: 29%; International Cancer Genome Consortium: 23%) [3, 13]. Indeed, the frequency of *PIK3CA* amplification is the highest among the aberration frequencies of key PI3K pathway members including *PTEN*, *PIK3R1*, *PIK3R2*, and *AKT* in serous ovarian cancers. On the contrary, *PIK3CA* amplification was not detected in two patient cohorts of clear cell or mucinous subtype [14, 15].

Whether *PIK3CA* amplification causes comparable downstream effects to *PIK3CA* driver mutations in cancers is unclear. *PIK3CA* overexpression induced AKT phosphorylation in head and neck squamous carcinoma (HNSCC) cells [16], but AKT signaling was not activated upon *PIK3CA* overexpression in human nontumorigenic mammary cells or breast cancer cells [17, 18, 19], suggesting that increased *PIK3CA* expression may not necessarily activate AKT. It was demonstrated that *PIK3CA* copy number positively correlated with p110α kinase activity in ovarian cancer cell lines [20]. However, the phenotypic consequences and downstream signaling of *PIK3CA* amplification in serous ovarian cancer remain unresolved.

In this study, through extensive functional characterization of *PIK3CA*‐overexpressing or E545K CRISPR knock‐in serous ovarian cancer cells, we provide evidence that both of these two *PIK3CA* aberrations can promote ovarian tumorigenicity and activate AKT to induce downstream signaling pathways. Remarkably, apart from p110α‐specific or AKT inhibitor, N‐cadherin antagonist has shown potential for the treatment of *PIK3CA*‐aberrated ovarian cancer cells.

## 2 Materials and methods

### 2.1 Cell lines and stable cell line construction

Human ovarian cancer cell lines, including Caov3 (RRID: CVCL_0201), DOV13 (RRID: CVCL_6774), OVCAR5 (RRID: CVCL_1628), OVCAR8 (RRID: CVCL_1629), and HEYA8 (RRID: CVCL_8878), were obtained from National Cancer Institute (Bethesda, MD, USA) and maintained in RPMI1640 (Thermo Fisher Scientific, Waltham, MA, USA). HEK293‐FT cells (RRID: CVCL_6911) (from Thermo Fisher Scientific) were maintained in DMEM (Thermo Fisher Scientific). All culture media were supplemented with 5% fetal bovine serum (Thermo Fisher Scientific) and 100 units·mL^−1^ penicillin–streptomycin (Thermo Fisher Scientific). All cell lines were cultured at 37 °C with 5% CO_2_ in a humidified incubator. They were authenticated by STR analysis and validated as mycoplasma‐free. To generate lentivirus for establishing stable cell lines, HEK293‐FT cells (1 × 10^6^) seeded in 6‐well plates were transfected with 2 μg expression plasmids and lentivirus packaging plasmids (1.5 μg psPAX2 and 0.5 μg pMD2.G) using Lipofectamine 3000 (Thermo Fisher Scientific). The culture media were collected 48 and 72 h post‐transfection and were filtered using 0.45 μm filter. Ovarian cancer cells were transduced with the virus in the presence of 10 μg·mL^−1^ polybrene (Santa Cruz Biotechnology, Dallas, TX, USA) for 72 h. The cells were then selected with puromycin.

### 2.2 Plasmids

pHAGE‐*PIK3CA* was kindly provided by G. Mills and K. Scott (plasmid #116771; Addgene, Watertown, MA, USA) [21]. pHAGE‐GFP was used as a control vector. EFSp‐GFP‐Empty, EFSp‐GFP‐YAP, and EFSp‐GFP‐YAP (5SA) (5 LATS phosphorylation sites mutated to Ala) were gifts from R. Bremner (plasmids #174171, #174168 and #174170; Addgene) [22]. EFSp‐GFP‐YAP (S127A) and EFSp‐GFP‐YAP (S127D) were generated from EFSp‐GFP‐YAP using QuikChange Lightning Site‐Directed Mutagenesis Kit (Agilent Technologies, Santa Clara, CA, USA). All plasmids were validated by sequencing.

### 2.3 Generation of CRISPR/Cas 9‐based *PIK3CA* E545K knock‐in cells

*PIK3CA* E545K knock‐in cells were established using Alt‐R clustered regularly interspaced short palindromic repeats (CRISPR)‐Cas9 system based on manufacturer's instructions (IDT, Coralville, IA, USA). The sequences of crRNA and the single‐stranded donor template are shown in Table S1. Briefly, 1 μm crRNA‐tracrRNA complex was generated by mixing 1 μL of 100 μm crRNA (IDT), 1 μL of 100 μm ATTO 550‐labeled tracrRNA (IDT), and nuclease‐free water, followed by boiling at 95 °C for 5 min. CRISPR ribonucleoprotein (RNP) complex (150 μL) was then prepared by mixing 1 μm crRNA‐tracrRNA complex, 1 μm Cas9 enzyme (IDT), 7 μL Cas9 plus reagent (Thermo Fisher Scientific), and 107 μL Opti‐MEM medium (Thermo Fisher Scientific). OVCAR8 cells (2 × 10^5^) were transfected with the CRISPR RNP complex and the single‐stranded donor template at a ratio of 1 : 1.77 using Lipofectamine CRISPRMAX Transfection Reagent (Thermo Fisher Scientific). To increase the efficiency of homology directed repair (HDR), 5 μm of Alt‐R HDR enhancer (IDT) was added. The cells were then sorted for single cell 24 h post‐transfection, expanded, and verified by Sanger sequencing. Unedited cells which retained wild‐type *PIK3CA* alleles after the procedure served as control.

### 2.4 siRNA transfection

Cells (1 × 10^5^) were seeded in 6‐well plates and transfected with 10 nm siRNA using 3 μL lipofectamine RNAiMAX (Invitrogen, Waltham, MA, USA). The siRNA in 100 μL of Opti‐MEM medium was mixed with lipofectamine RNAiMAX in 100 μL of Opti‐MEM medium at room temperature for 10 min before adding to the cells. The cells were then harvested for subsequent assays 72 h after siRNA transfection. The sequences of the siRNA used are shown in Table S2.

### 2.5 Cell viability assay

Cells were seeded at a density of 1000 cells per well in 96‐well plates in triplicate. At the indicated time points, the culture medium was aspirated and replaced by new medium with 10% (v/v) 0.2 mg·mL^−1^ resazurin (Sigma‐Aldrich, St. Louis, MO, USA). After 4 h of incubation at 37 °C in the dark, fluorescence signal was measured at excitation and emission wavelengths of 570 and 600 nm, respectively, using a microplate reader (Varioskan Flash; Thermo Fisher Scientific). Relative cell viability was obtained after subtracting the average reading of the blanks.

### 2.6 Colony formation assay

Cells were seeded at a density of 1000 cells per well in 6‐well plates. After 8 days, cell colonies were stained with 0.5% crystal violet dissolved in 20% ethanol for 10 min and then rinsed with PBS.

### 2.7 Cell migration or invasion assay

Cell suspension (1.2 × 10^4^) in serum‐free medium was seeded into 8‐μm inserts (Millipore, Billerica, MA, USA) coated with 1 mg·mL^−1^ Matrigel (Corning, Glendale, AZ, USA) (for invasion assay) or uncoated inserts (for migration assay). RPMI1640 with 10% FBS was added to the lower chamber as a chemoattractant. Cells migrated or invaded through the insert membrane after 16 h at 37 °C were fixed with ice‐cold methanol for 30 min and stained with 0.5% crystal violet for 20 min. Five random representative images (200×) were captured for each insert using (Olympus IX71, Shinjuku‐ku, Tokyo, Japan) inverted microscope with Olympus DP71 color digital camera. The number of migrated or invaded cells was quantified using (imagej, National Institutes of Health, Bethesda, MD, USA).

### 2.8 Scratch assay

To measure cell migration across a scratch‐induced gap *in vitro*, linear wounds were made in cell monolayers in 24‐well plates using 200 μL pipette tip. The cells were cultured in serum‐free medium and the wounds were visualized using Olympus IX71 inverted microscope 0 and 16 h after the scratch was made. Alternatively, live‐cell migration was photographed using an IN Cell Analyzer 6500HS System (Cytiva, Amersham, UK) at 37 °C with 5% CO_2_ in a humidified incubator 0, 4, 8, 16, and 20 h after scratch. The wound areas were analyzed from microscopic images using imagej and were represented graphically as percentage of wound closure.

### 2.9 Cell cycle assay

Cells were plated at a density of 2 × 10^5^ cells in 60 mm dishes. On the next day, the cells were treated with 2 mm thymidine (Sigma‐Aldrich) for synchronization and incubated at 37 °C for 16 h. After being released in fresh media for 8 h, the cells were synchronized with 2 mm thymidine again for another 16 h and collected at the indicated time points. The cells were washed with ice‐cold PBS twice and fixed with ice‐cold 70% ethanol overnight. To measure DNA content, the fixed cells were treated with 100 μL RNase A solution (50 μg·mL^−1^; Sigma‐Aldrich) for 30 min at 37 °C and then subjected to propidium iodide staining (20 μg·mL^−1^; Sigma‐Aldrich). The samples were analyzed on NovoCyte Advanteon BVYG using NovoExpress software (Agilent Technologies). About 10 000 events were assessed per measurement.

### 2.10 *In vivo* tumorigenic assay

All animal procedures were approved by the Committee on the Use of Live Animals in Teaching and Research at the University of Hong Kong (protocol number: 5565‐20). The experiments were performed in accordance with the approved protocol and institutional regulations. Cells (5 × 10^6^) were suspended in sterile PBS and implanted via intraperitoneal (i.p.) injection into the 6‐week‐old female athymic nude mice (BALB/cAnN‐nu; *n* = 5 per group) (Charles River Lab, Stone Ridge, NY, USA), which were housed in individually ventilated cages inside environmentally controlled rooms under 12‐h light/dark cycle and with free access to water and food. After 6 weeks, mice were sacrificed and the disseminated tumor nodules and ascites in peritoneal cavity were collected and weighed. Large tumor nodules were fixed in 4% paraformaldehyde and paraffin‐embedded for histology and immunohistochemistry. In the treatment experiments, 2 weeks after the cells were injected, mice (*n* = 5 per group) were randomly assigned to receive one of the following treatments 5 days per week for 3 weeks: (a) vehicle, (b) alpelisib, (c) ADH‐1, (d) alpelisib and ADH‐1 combination. Alpelisib was dissolved in 0.5% methylcellulose in saline and administered via oral gavage at 25 mg·kg^−1^. ADH‐1 was dissolved in 5% DMSO + 40% PEG300 + 5% Tween 80 in saline and administered via i.p. at 25 mg·kg^−1^. The mice were weighed throughout the study. The disseminated tumor nodules and ascites in the peritoneal cavity were collected and measured after sacrifice.

### 2.11 Drug sensitivity assay

Cells (1000/well) were seeded in triplicate in 96‐well plates. The cells were treated with the inhibitors listed in Table S3 at serially diluted concentration for 72 h prior to measurement of cell viability. In scratch assay for cell migration, cells seeded in 24‐well plates were treated with the inhibitors for 24 h.

### 2.12 Western blotting

Cells were lysed in RIPA buffer (1% sodium deoxycholate, 1% NP‐40, 150 mm NaCl, 0.1% sodium dodecyl‐sulfate (SDS)) supplemented with protease and phosphatase inhibitors (Thermo Fisher Scientific). Protein samples mixed with 6× SDS loading buffer (25% glycerol, 62.5 mm Tris/HCl, pH 6.8, 2% SDS, 0.6 m DTT, 0.01% bromophenol blue) were subjected to SDS‐polyacrylamide gel electrophoresis and transfer to methanol‐activated polyvinylidene fluoride (PVDF) membrane (Millipore). The PVDF membrane was then blocked with 5% nonfat milk in 1 × Tris‐buffered saline with Tween 20 buffer (150 mm NaCl, 20 mm Tris, pH 7.5, 0.1% Tween 20) at room temperature, followed by primary antibody incubation at 4 °C overnight and horseradish peroxidase (HRP)‐conjugated secondary antibody incubation for another 2 h at room temperature. The protein was detected using electrochemiluminescence kit (Bio‐Rad, Hercules, CA, USA). The primary and secondary antibodies used in this study are shown in Table S4. ERK2 was used as loading control. Densitometry analysis of the western blots was performed using imagej.

### 2.13 Reverse phase protein array

Reverse phase protein array (RPPA) was conducted by the MD Anderson Cancer Center RPPA Core Facility. Cells were lysed by RIPA buffer supplemented with freshly added proteinase and phosphatase inhibitor. Protein lysates (two biological replicates) were adjusted with the lysis buffer and 4× SDS sample buffer (8% SDS, 0.25 m Tris/HCl, pH 6.8, 40% glycerol, 10% freshly added β‐mercaptoethanol) to 1.5 μg·μL^−1^. The denatured samples were then printed on nitrocellulose‐coated slides and incubated with primary and secondary antibodies. Signals from HRP and 3,3′‐diaminobenzidine (DAB; Amresco, Solon, OH, USA) colorimetric reaction were quantitated using rppa space software [23].

### 2.14 Subcellular fractionation

The Minute Cytoplasmic & Nuclear Extraction Kits for Cells (Invent Biotechnologies, Plymouth, MN, USA) was used to isolate cytoplasmic and nuclear proteins from total cell lysates. Cells were first lysed in the Cytoplasmic Extraction Buffer to obtain the cytoplasmic fraction from the supernatant after vortex and centrifugation. The remaining pellet was resuspended in nuclear extraction buffer to obtain nuclear proteins.

### 2.15 RNA sequencing

Total RNA (three biological replicates per condition) was extracted using TRIzol reagent (Thermo Fisher Scientific). Poly(A)‐seq library preparation, RNA sequencing (RNA‐seq), and subsequent data processing were performed at Novogene (Beijing, China). Briefly, libraries were prepared using NEBNext^®^ Ultra™ RNA Library Prep Kit for Illumina^®^ (New England Biolabs, Ipswich, MA, USA). Sequencing was performed on Illumina NovaSeq 6000 system (Illumina, San Diego, CA, USA). Raw reads were filtered for adapter sequence, low‐quality sequence, and rRNA sequence. The retained reads were aligned to the human genome GRCh38 using (star aligner version 2.7.3a, Cold Spring Harbor Laboratory, Cold Spring Harbor, NY, USA). Differentially expressed genes (DEGs; *PIK3CA* overexpression vs empty vector or *PIK3CA* E545K mutation vs control) were identified using (deseq2 version 1.26.0, Illumina, San Diego, CA, USA). One hundred and seventy‐two DEGs from the intersection of three sets (*PIK3CA* overexpression vs empty vector, *PIK3CA* E545K homozygous vs control, and *PIK3CA* E545K heterozygous vs control) with adjusted *P*‐value < 5% were subjected to gene ontology (GO) enrichment analysis using (metascape https://metascape.org). Gene expression data of *PIK3CA* E545K mutation and corresponding control were subjected to gene set enrichment analysis (GSEA) on the hallmark gene sets using (gsea version 4.3.2, Broad Institute, Cambridge, MA, USA).

### 2.16 Real‐time PCR

Total RNA (1 μg) was used for reverse transcription by the HiScript II 1st Strand cDNA Synthesis Kit (Vazyme, Nanjing, China). The newly synthesized cDNA was diluted 5‐fold. Real‐time PCR was performed in triplicate with ChamQ SYBR Color qPCR Master Mix (Vazyme) on a CFX Opus 96 Real‐Time PCR detection system (Bio‐Rad). The relative expression of mRNA was determined by the ΔΔ*C*_t_ method, where *C*_t_ was referred as threshold cycle, and *GAPDH* was used for normalization. Sequences of the primers used are listed in Table S5.

### 2.17 Immunofluorescence

Cells (1.6 × 10^5^) were seeded onto sterile cover slips in 6‐well plate and cultured at 37 °C overnight. Adhered cells were then fixed with 4% paraformaldehyde, followed by permeabilization with 0.1% Triton X‐100 on ice for 10 min. To reduce the nonspecific binding of the antibodies, the cells were blocked with 3% BSA at room temperature for 30 min. After overnight incubation with primary antibodies at 4 °C, the cells were rinsed with PBS for three times and subjected to secondary antibody incubation for 1 h at room temperature. The cells were incubated with a mounting medium containing DAPI to stain the nuclei. Images were then taken with Carl Zeiss LSM 710 confocal microscope (Zeiss, Oberkochen, Germany) and analyzed by imagej. The nuclear/cytoplasmic ratio was calculated by dividing the mean fluorescence intensity in the nucleus by the mean fluorescence intensity in the cytoplasm.

### 2.18 Immunohistochemistry

Immunohistochemical staining was performed using human ovarian cancer tumor tissue array (OVC1021; Pantomics, Inc., Richmond, CA, USA) or harvested tumors from xenografts. The tissue sections were deparaffinized and rehydrated followed by antigen retrieval using citrate buffer pH 6.0. The slides were incubated with 3% H_2_O_2_ to reduce endogenous peroxidase activity and 10% BSA to block unspecific binding, prior to incubation with anti‐p110α, anti‐YAP pS127, or anti‐N‐cadherin antibody overnight at 4 °C and biotin‐conjugated secondary antibody (Dako, Carpinteria, CA, USA) for 1 h in dark at room temperature. DAB was used to detect signal from HRP. Immunoreactive score (IRS) was calculated by multiplying the score of percentage of positive cells (0, negative; 1, < 10%; 2, 10–50%; 3, 51–80% and 4, > 80%) and the intensity score (0 = negative, 1 = mild, 2 = moderate, and 3 = intense). IRS on an arbitrary scale: 0–1, no immunoreactivity; 2–3, mild; 4–8, moderate; and 9–12, strongly positive were used to represent protein expression levels.

### 2.19 Luciferase assay

The 8×GTIIC‐luciferase reporter construct (8×GTIIC‐luc) was kindly provided by S. Piccolo (plasmid #34615; Addgene) [24]. Cells (triplicate per condition) were co‐transfected with 8×GTIIC‐luc or pGL3 basic reporter vector with pRL‐TK Renilla luciferase plasmid using Lipofectamine 3000. After 48 h, firefly and Renilla luciferase activities were measured using Dual‐Luciferase^®^ Reporter Assay System (Promega, Madison, WI, USA) and Varioskan™ LUX multimode microplate reader (Thermo Fisher Scientific).

### 2.20 Active RAC1 detection assay

RAC1 activity was measured using RAC1 Activation Assay Kit (Sigma‐Aldrich) which involves immunoprecipitation of active RAC1 and subsequent detection. Cells were lysed in Mg^2+^ lysis/wash Buffer containing 10% glycerol and protease and phosphatase inhibitors prior to incubation with glutathione agarose (Santa Cruz Biotechnology) for 10 min at 4 °C for preclearing. The lysates were then gently rocked with PAK1 PBD‐agarose beads at 4 °C for another 60 min. The bead‐immobilized GTP‐bound RAC1 protein was eluted from the beads by boiling in 2× Laemmli sample buffer at 95 °C for 10 min and was detected by western blotting.

### 2.21 Patient survival analysis and *PIK3CA* copy number analysis

Data from Kaplan–Meier (KM) Plotter online database (https://kmplot.com) [25] were analyzed for patient survival based on the mRNA levels of *PIK3CA* (detected by probe 204369_at) or that of *CDH2* (probe 203440_at). The TCGA ovarian cancer dataset downloaded from the Broad GDAC Firehose (http://gdac.broadinstitute.org/) was used for analysis involving *PIK3CA* copy numbers.

### 2.22 Statistical analysis

All experiments were performed three times. All data in this study were represented as mean ± standard deviation. (graphpad prism 10, GraphPad Software, San Diego, CA, USA) was utilized to perform statistical analyses. Unless otherwise stated, statistical analysis was done by two‐tailed Student's *t*‐test or ANOVA. Statistical significance of linear correlation analysis was evaluated using Pearson's correlation coefficient. *P*‐values < 0.05 were considered statistically significant.

## 3 Results

### 3.1 *PIK3CA* amplification and hotspot mutation E545K promote tumorigenic phenotypes of ovarian cancer

Increased *PIK3CA* copy number (gain or amplification represented by GISTIC annotation of +1 or +2) was detected in 79.4% of TCGA serous ovarian tumor samples (*n* = 398) (Fig. S1A). There was a marked correlation between *PIK3CA* copy number and its mRNA levels (*r* = 0.61, *P* < 0.0001), which in turn positively correlated with p110α protein levels (*r* = 0.57, *P* < 0.0001) (Fig. S1B), suggesting *PIK3CA* copy number as an influence on gene and protein expression. High *PIK3CA* mRNA level was significantly associated with poor survival in ovarian cancer patients in KM plotter dataset (patients divided at median, tertile, or quartile) (Fig. S1C).

**Fig. 1:**
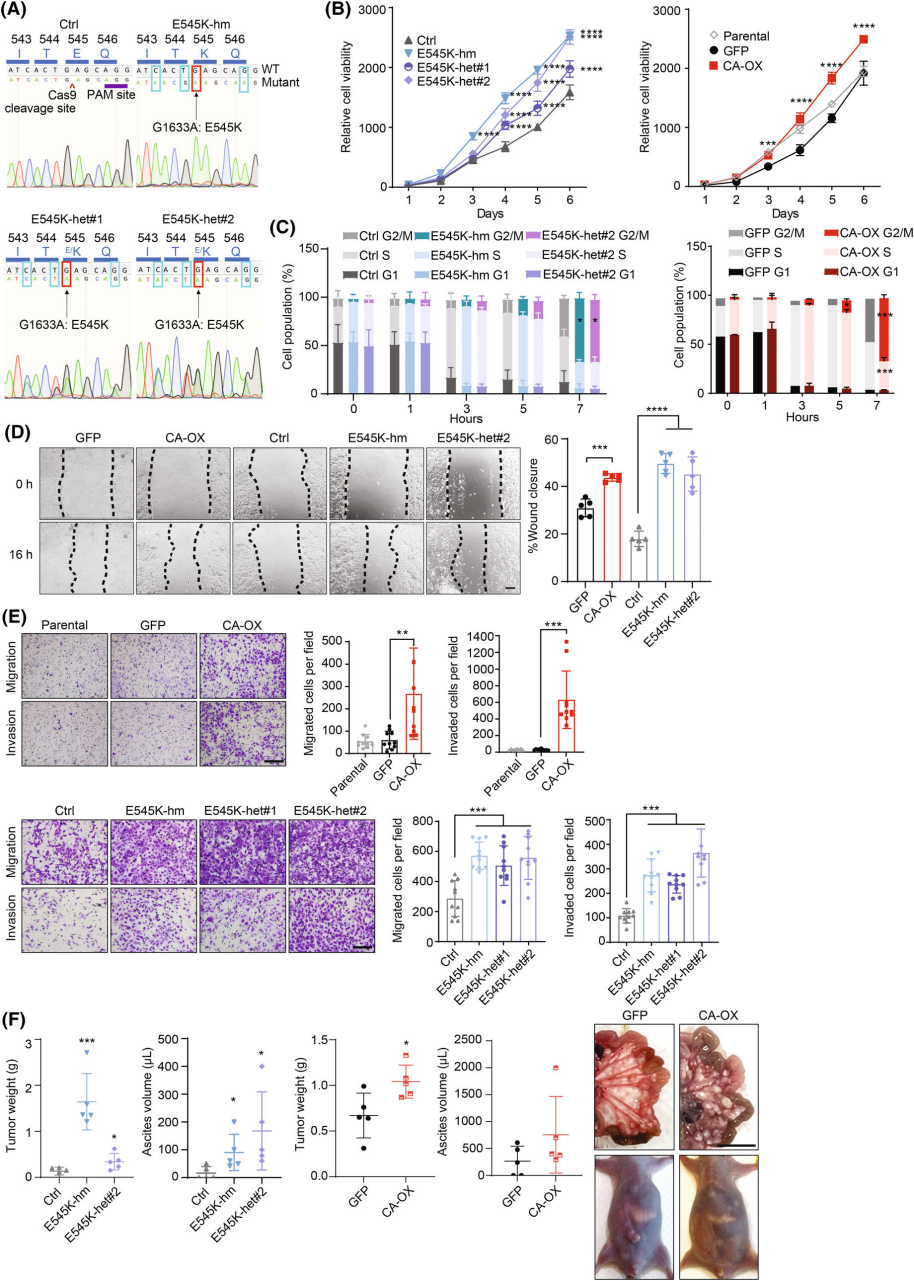
*PIK3CA* aberrations induced tumorigenic phenotypes of ovarian cancer cells. (A) Sanger sequencing chromatograms of the unedited clone (Ctrl), a homozygous *PIK3CA* E545K CRISPR knock‐in clone (hm), and two heterozygous *PIK3CA* E545K CRISPR knock‐in clones (het#1 and het#2). Red box, E545K mutation; cyan box, silence mutation within the target sequence and the protospacer adjacent motif (PAM) to avoid re‐cutting of the repair template. (B) Viability of the *PIK3CA* E545K knock‐in OVCAR8 cells and the unedited control (left) or OVCAR8 cells with stable overexpression (OX) of wild‐type *PIK3CA* or GFP vector (right) was measured at the indicated time points (*n* = 3). (C) The CRISPR knock‐in OVCAR8 cells (left), OVCAR8 cells with stable overexpression of wild‐type *PIK3CA* (right) and their respective controls were synchronized by double thymidine block and harvested at the indicated postrelease time points for cell cycle analysis (*n* = 3). (D) Scratch assay of *PIK3CA*‐overexpressing OVCAR8 cells and *PIK3CA* E545K knock‐in OVCAR8 cells. Images at 100× were taken at 0 and 16 h after scratch was made. Scale bar, 200 μm. Percentages of wound closure of five fields are presented in bar graph. (E) The OVCAR8 cells were seeded into the inserts without (migration assay) or with (invasion assay) Matrigel coating and harvested after 16 h of incubation. Representative images at 200× are shown. Scale bar, 200 μm. Numbers of migrated/invaded cells from 10 fields are presented in bar graphs. (F) The OVCAR8 cells were intraperitoneally injected into nude mice (*n* = 5) for 8 weeks. Tumor weight and ascites volume (left) and representative images of tumor nodules and abdomen expansion (right) are shown. Scale bar, 1 cm. All the graphs display the mean value ± SD. *P < 0.05; **P < 0.01; ***P < 0.001; ****P < 0.0001 compared with control or vector using two‐way ANOVA (B, C) or one‐way ANOVA (D–F) with Sidak's multiple comparison test or *t*‐test (F, GFP vs CA‐OX).

To investigate the functional consequences of *PIK3CA* amplification, serous ovarian cancer cells with stable wild‐type *PIK3CA* overexpression, which served as a mimic of *PIK3CA* gene amplification, were generated by lentiviral transduction. OVCAR8 and DOV13 cells are chosen because they carry relatively low *PIK3CA* copy numbers and p110α protein levels [26] (Fig. S2A) and express wild‐type PI3K pathway members in particular *PIK3CA*. The overexpression was confirmed by western blotting (Fig. S2B). Importantly, p110α protein levels in these overexpression cell lines were comparable to that in cell lines with intrinsic *PIK3CA* gene amplification including CaOV3, OVCAR5, and HEYA8 (Fig. S2C). We also used CRISPR to generate *PIK3CA* E545K knock‐in OVCAR8 cells, which were confirmed by Sanger sequencing (Fig. 1A). The two heterozygous clones (one of the alleles mutated; denoted as het#1 and het#2), one homozygous clone with both alleles mutated (denoted as hm) and the unedited control (cells retained wild‐type *PIK3CA* alleles after the same editing procedure) had similar p110α protein levels as the parental cells (Fig. S2D).

Consistent with the known oncogenicity, the homozygous or heterozygous E545K mutation enhanced cell viability and colony formation (Fig. 1B; Fig. S2E). Interestingly, these enhanced phenotypes could also be observed in wild‐type *PIK3CA*‐overexpressing OVCAR8 or DOV13 cells (Fig. 1B; Fig. S2E,F). These cells showed faster exit from S phase to G2/M phase (Fig. 1C). Additionally, the *PIK3CA*‐overexpressing cells and E545K mutant cells all displayed enhanced abilities in cell migration and invasion (Fig. 1D,E). To assess tumorigenesis *in vivo*, BALB/c female nude mice were i.p. injected with the OVCAR8 cells. Ovarian cancer metastasis is characterized by peritoneal dissemination and ascites formation. The i.p. xenografts derived from E545K mutant cells or *PIK3CA*‐overexpressing cells developed more metastatic implants on the peritoneal surface, omentum, and mesentery compared with the controls. As shown in Fig. 1F, E545K knock‐in resulted in significantly more metastases as gauged by weight of tumor nodules and ascites volume compared with control (*P* < 0.05). Mice bearing *PIK3CA*‐overexpressing OVCAR8 cells also showed increased tumor implants (*P* < 0.05) and ascites (although not statistically significant) (Fig. 1F). Collectively, the promotion of tumorigenic phenotypes by wild‐type *PIK3CA* and the E545K hotspot mutation suggested that the two types of *PIK3CA* aberrations (amplification and mutation) carry oncogenic roles in serous ovarian cancer.

### 3.2 *PIK3CA* amplification activates AKT signaling but not mitogen‐activated protein kinase (MAPK)

**Fig. 2:**
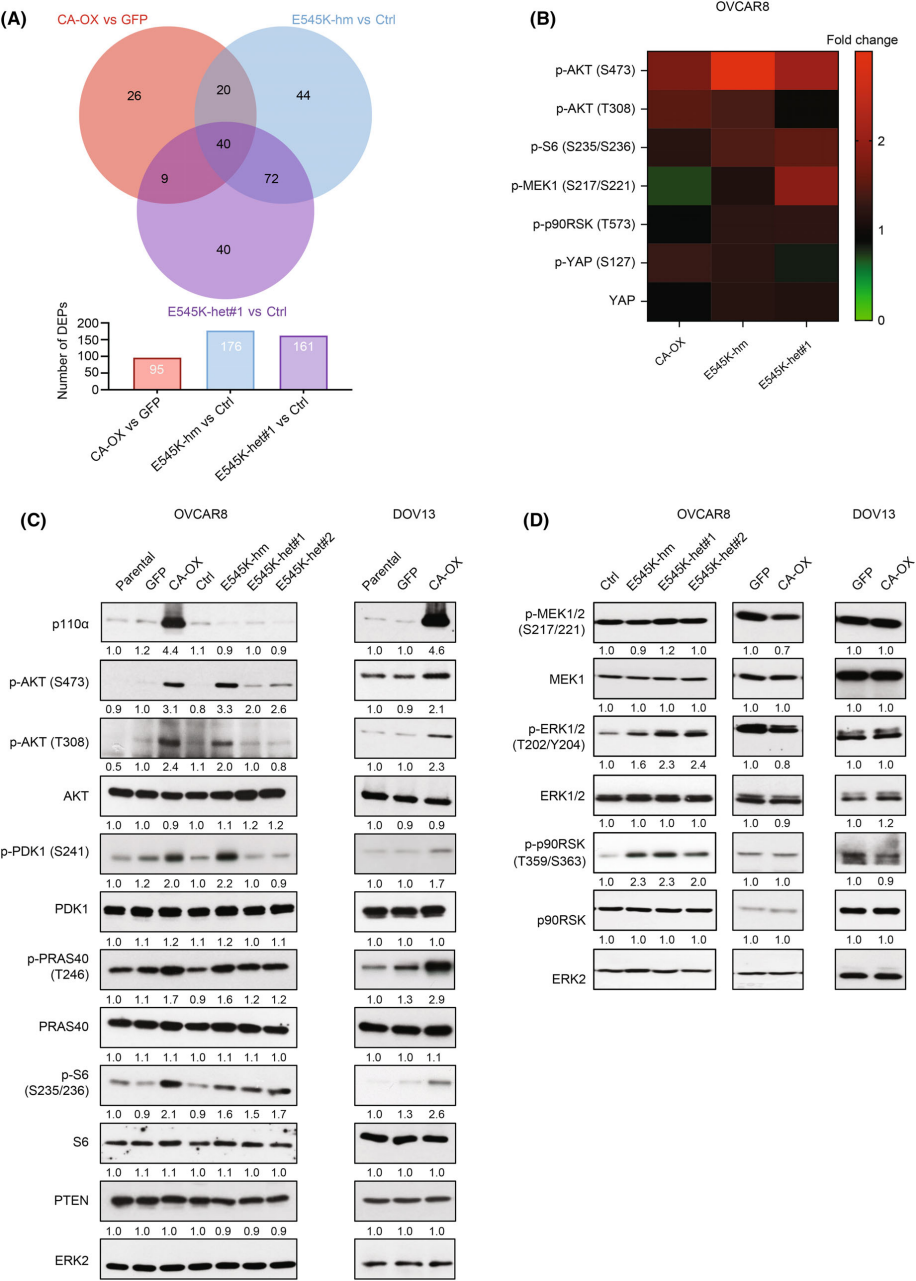
AKT signaling pathway was activated by both *PIK3CA* amplification and E545K mutation, whereas MAPK signaling pathway was selectively induced by E545K. (A) Protein lysates of the OVCAR8 wild‐type *PIK3CA*‐overexpressing cells (CA‐OX), E545K knock‐in cells (homozygous knock‐in clone (hm), heterozygous knock‐in clone (het#1)), and their corresponding controls were harvested for reverse phase protein array analysis (*n* = 2). The Venn diagram shows the distribution of the differentially expressed proteins (DEPs) (fold change > 1.2, *P* < 0.05). (B) The heatmap illustrates the alterations in the selected protein expression upon *PIK3CA* overexpression or E545K mutation. (C, D) Lysates of OVCAR8 E545K knock‐in cells, OVCAR8 *PIK3CA*‐overexpressing cells, and DOV13 *PIK3CA*‐overexpressing cells were subjected to western blotting for proteins in the (C) AKT and (D) MAPK signaling pathways (*n* = 3). ERK2 was used as loading control. Numbers below blots are densitometric values normalized to that of ERK2 relative to the control.

To identify the potential tumor‐promoting mechanisms by the *PIK3CA* aberrations, we examined the differences in cancer signaling pathways between E545K knock‐in cells, wild‐type *PIK3CA*‐overexpressing cells and their corresponding controls using protein array. There were 40 differentially expressed proteins (DEPs; defined as fold change > 0.2 at *P* < 0.05) commonly identified in all *PIK3CA*‐aberrated cells (Fig. 2A). Among them were major members of the PI3K/AKT pathway (Fig. 2B). Subsequent western blotting confirmed that both *PIK3CA* E545K knock‐in cells and *PIK3CA*‐overexpressing cells demonstrated higher phosphorylation levels of AKT, PRAS40, and ribosomal S6, compared with the controls (Fig. 2C). This demonstrated the ability of the *PIK3CA* aberrations in activating the AKT pathway in serous ovarian cancer cells. Intriguingly, the increases in AKT phosphorylation at Ser473 (at maximum of 3.3‐fold) appeared to be more robust than that at Thr308 (at maximum of 2.4‐fold) in both protein array and western blot data of OVCAR8. Further, AKT Thr308 and PDK1 Ser241 phosphorylation (1.7‐ to 2.2‐fold increase) were only induced in the homozygous E545K cells and *PIK3CA*‐overexpressing cells but not in the two heterozygous E545K cells (Fig. 2C). Indeed, Thr308 and Ser473 phosphorylation were activated by different upstream molecules, with Thr308 phosphorylation activated by PDK1 and Ser473 phosphorylation by mTORC2. Hence, the synchronized trend in the phosphorylation of Thr308 and PDK1 we observed may be reasonable. Yet, whether this difference in AKT phosphorylation leads to any functional significance awaits further investigation. The tumor suppressor and major negative regulator of the PI3K pathway, PTEN, was unaltered in these cells (Fig. 2C).

It has been reported that *PIK3CA* E545K activates the MAPK pathway in human breast epithelial cells [27]. Consistently, the protein array data and western blots showed that the phosphorylation levels of ERK1/2 and p90RSK were upregulated in the E545K mutant cells (Fig. 2B,D). The protein array data suggested upregulation of p‐MEK1 in the E545K mutant cells. However, this could not be verified by the western blots as there were no changes observed. OVCAR8 or DOV13 cells with *PIK3CA* overexpression did not exhibit increase in any of these MAPK pathway members, indicating that *PIK3CA* amplification does not activate ERK1/2 signaling as the E545K mutation.

### 3.3 *PIK3CA* overexpression sensitizes serous ovarian cancer cells to p110α‐specific inhibitor and AKT inhibitor but not MEK inhibitor

**Fig. 3:**
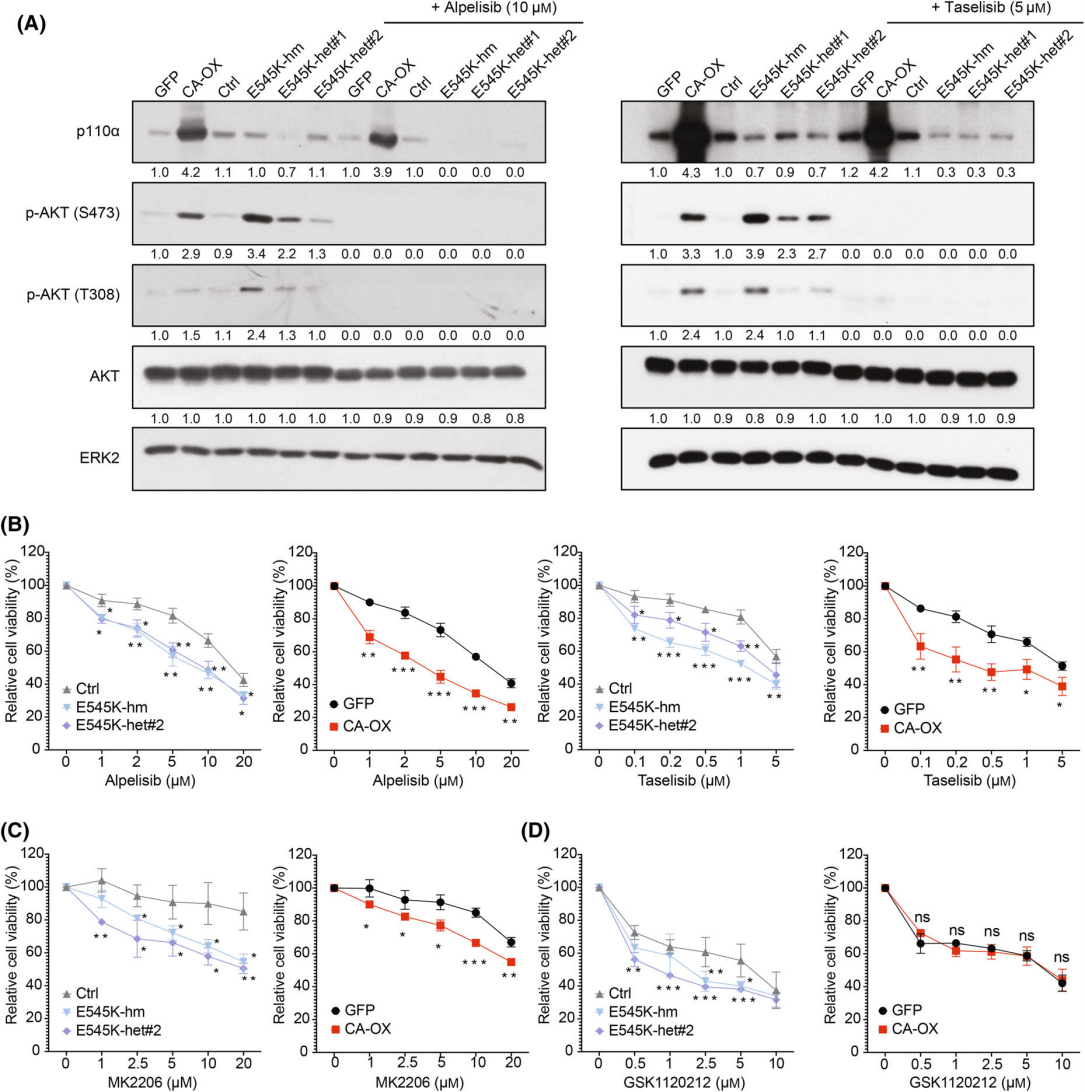
*PIK3CA* aberrations conferred higher sensitivity towards p110α or AKT inhibitors. (A) OVCAR8 wild‐type *PIK3CA*‐overexpressing cells (CA‐OX), E545K knock‐in cells (homozygous knock‐in clone (hm), heterozygous knock‐in clones (het#1 and het#2)), and their corresponding controls were treated with the indicated concentration of alpelisib or taselisib for 48 h prior to western blotting (*n* = 3). Numbers below blots are densitometric values normalized to that of ERK2 relative to the control. (B–D) Dose–response curves showing the sensitivity of the indicated *PIK3CA*‐aberrated cells towards 72‐h treatment with (B) alpelisib or taselisib; (C) MK2206; or (D) GSK1120212. Data shown are mean ± SD (*n* = 3). *P < 0.05; **P < 0.01; ***P < 0.001; ns, no significant difference by two‐way ANOVA with Sidak's multiple comparison test.

Alpelisib (BYL719) is a potent p110α‐specific inhibitor that has a dual mechanism of action by inhibiting p110α kinase activity and triggering the degradation of p110α mutant protein [28]. The majority of the previous studies reported the effects of alpelisib in *PIK3CA*‐mutant cells. Whether *PIK3CA* amplification renders cancer cells sensitive to p110α inhibition was not clear. Our data showed that while the expression level of the p110α E545K mutant protein was markedly reduced upon treatment with alpelisib, p110α level in wild‐type *PIK3CA*‐overexpressing cells was unaltered (remained as 4‐fold compared with the controls; Fig. 3A). Nonetheless, alpelisib effectively suppressed AKT phosphorylation (Thr308 and Ser473) in all these *PIK3CA*‐aberrated cells. Drug response assays revealed that the E545K mutant or *PIK3CA*‐overexpressing cells were significantly more susceptible to alpelisib compared with the control cells (Fig. 3B). Taselisib (GDC‐0032) is a p110β‐sparing inhibitor and depletes p110α mutant protein [29, 30]. Similar to alpelisib, taselisib was more potent in *PIK3CA*‐aberrated cells than control cells (Fig. 3B). These data demonstrated that *PIK3CA* amplification can indicate sensitivity to p110α inhibition.

We also evaluated the inhibitory effects of AKT or MEK inhibitor. Higher sensitivity towards AKT inhibitor (MK2206) or MEK inhibitor (GSK1120212) was observed in cells with *PIK3CA* E545K mutation than in the unedited control cells (Fig. 3C,D). Likewise, cells with wild‐type *PIK3CA* overexpression were more sensitive to AKT inhibition. However, these cells were not more sensitive to the MEK inhibitor compared with the control. These results were consistent with the signaling pathway activation status described above.

### 3.4 *PIK3CA* aberrations cause YAP phosphorylation at Ser127 through AKT

**Fig. 4:**
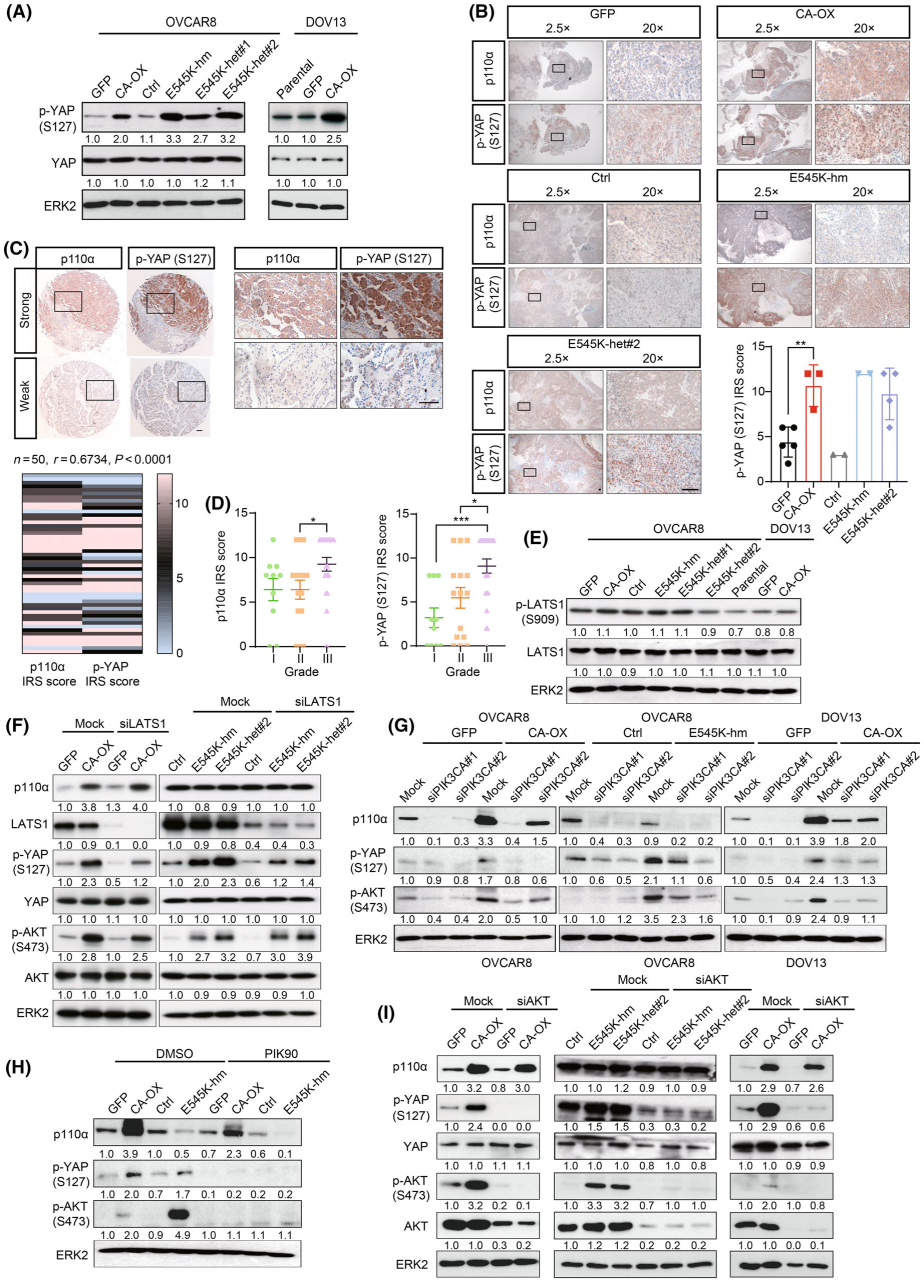
*PIK3CA* aberrations increased YAP Ser127 phosphorylation through p110α/AKT. (A) *PIK3CA* E545K knock‐in OVCAR8 cells (homozygous knock‐in clone (hm), heterozygous knock‐in clones (het#1 and het#2)), OVCAR8 or DOV13 cells with stable wild‐type *PIK3CA* overexpression (CA‐OX) were harvested for western blotting (*n* = 3). ERK2 was used as loading control. (B) Representative immunohistochemical images of xenograft tumors derived from *PIK3CA*‐overexpressing or *PIK3CA* E545K knock‐in cells stained with anti‐p110α antibody or anti‐p‐YAP (Ser127) at 2.5× and 20×. Quantification of staining using immunoreactive score (IRS) is shown in the bar chart at right corner (*n* = 2–5). Scale bar, 100 μm. (C) Human ovarian tumor tissue samples (*n* = 50) were subjected to immunohistochemical staining using anti‐p110α antibody or anti‐p‐YAP (Ser127). Top, representative immunohistochemical images. The boxes depict magnified areas. Scale bar, 100 μm. Bottom, heatmap illustrating the correlation between p110α and p‐YAP (Ser127) staining intensities. Pearson correlation (*r*) and *P*‐value of Pearson correlation analysis are shown. (D) IRS scores of p110α and p‐YAP (Ser127) stratified based on tumor grades of the patient samples analyzed in (C). (E) Lysates of *PIK3CA* E545K knock‐in OVCAR8 cells, OVCAR8 or DOV13 cells with stable wild‐type *PIK3CA* overexpression were subjected to western blotting (*n* = 3). (F–I) The cells were transfected with (F) *LATS1* siRNA, (G) *PIK3CA* siRNA for 72 h, (H) PIK90 treatment for 48 h, or (I) *AKT1*, *AKT2*, and *AKT3* siRNA for 72 h prior to western blotting (*n* = 3). All the graphs display the mean value ± SD. *P < 0.05; **P < 0.01; ***P < 0.001 using one‐way ANOVA with Sidak's multiple comparison test. Numbers below blots are densitometric values normalized to that of ERK2 relative to the control.

Intriguingly, the protein array data revealed an increased level of phosphorylated YAP at Ser127 in the presence of *PIK3CA* aberrations (Fig. 2B). Validation by western blotting showed a 2.0‐ to 3.3‐fold increase of YAP Ser127 levels compared with the controls (Fig. 4A). No significant difference was observed in total YAP protein level, suggesting that the induced phosphorylation was unlikely due to change in total protein expression. The higher levels of YAP Ser127 could also be observed in the metastatic tumors obtained from xenograft of *PIK3CA*‐overexpressing cells or E545K mutant OVCAR8 cells (Fig. 4B). It has been reported that high cell confluency induces YAP Ser127 phosphorylation [31]. In this regard, we kept the cells in sub‐confluent culture condition in all assays. We further evaluated the levels of p110α and YAP Ser127 in a set of serous ovarian cancer patient samples (*n* = 50) using immunohistochemistry (IHC). p110α protein expression was found to have a significant positive correlation with YAP Ser127 (*r* = 0.67; *P* < 0.0001) (Fig. 4C). Also, the histological grades of the tumors correlated with immunoreactivity of p110α or that of YAP Ser127 (Fig. 4D). Consistent with previous studies that YAP Ser127 phosphorylation leads to cytoplasmic retention of the protein [31], the staining was predominantly in the cytoplasm.

YAP Ser127 is a site known to be phosphorylated by the LATS1/2 kinases of the Hippo pathway [31]. However, we did not observe any change in the level of phosphorylated LATS1 or total LATS1 (Fig. 4E). Moreover, YAP Ser127 levels remained > 2‐fold higher in *PIK3CA*‐aberrated cells than the controls upon LATS1 or LATS2 depletion by siRNA (Fig. 4F; Fig. S3), suggesting that YAP Ser127 phosphorylation is unlikely mediated by LATS1/2 in these cells. YAP is also a reported substrate of AKT, with the Ser127 residue directly phosphorylated by AKT [32]. To evaluate if AKT is the upstream kinase of YAP Ser127 in *PIK3CA*‐aberrated cells, we inhibited the PI3K/AKT pathway through siRNA or small molecule inhibitor. Interestingly, the induction of YAP Ser127 phosphorylation in *PIK3CA*‐overexpressing or E545K mutant cells was consistently abrogated in the presence of *PIK3CA* siRNA, PI3K inhibitor (PIK90), or *AKT1/2/3* siRNA (Fig. 4G–I), suggesting that PI3K/AKT mediates YAP Ser127 phosphorylation.

### 3.5 *PIK3CA* aberrations retain YAP in the cytoplasm with promotion of cell migration and RAC1 activation

**Fig. 5:**
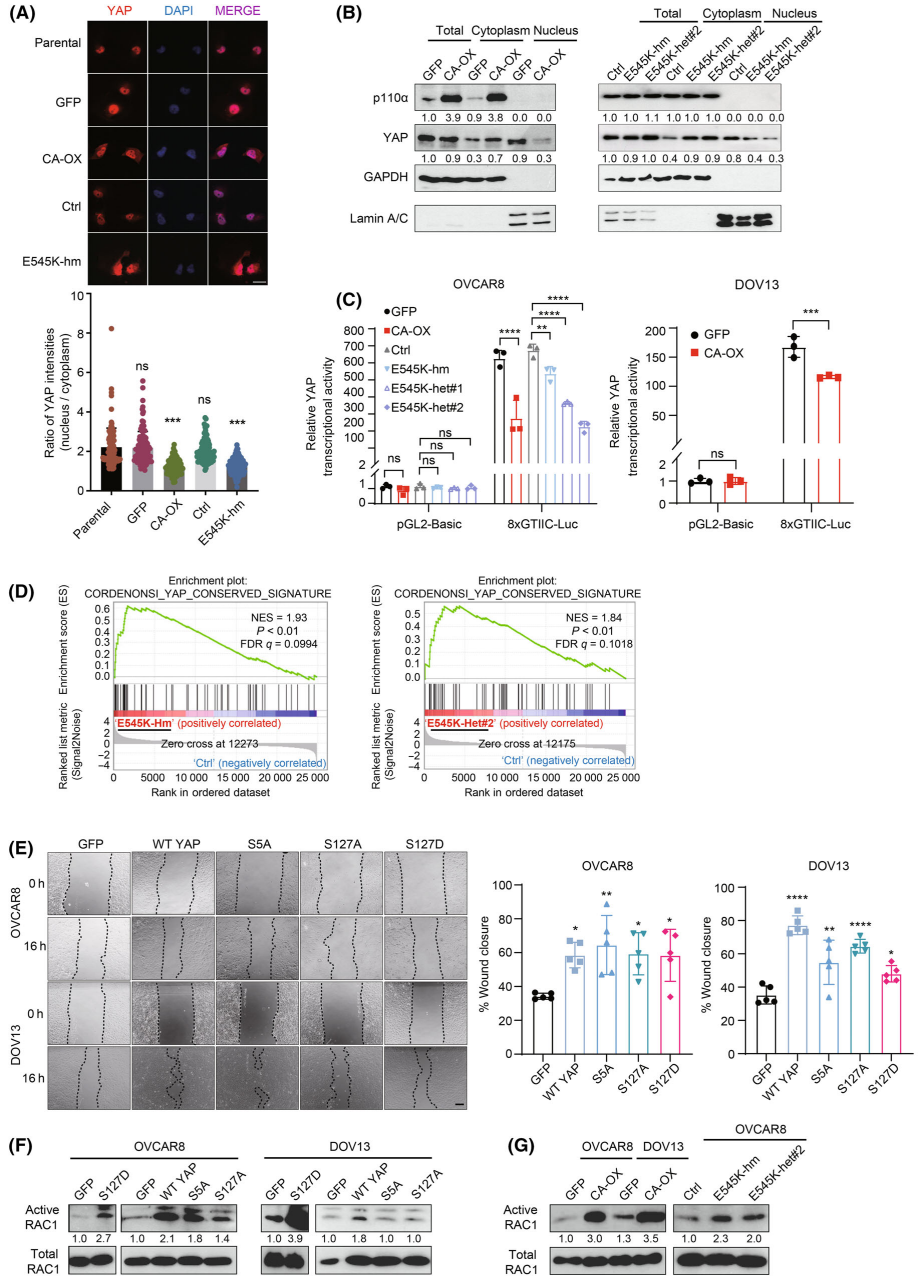
*PIK3CA* aberrations promoted YAP cytoplasmic retention to increase cell migration through active RAC1. (A) Representative immunofluorescence images showing YAP (red) in OVCAR8 cells with wild‐type *PIK3CA* overexpression (CA‐OX) or homozygous E545K knock‐in mutation (hm). Nuclei were stained with DAPI (blue). The nuclear/cytoplasmic intensity ratio of YAP in individual cell (*n* = 115) is presented in the bar chart. Scale bar, 20 μm. (B) Lysates of OVCAR8 cells with wild‐type *PIK3CA* overexpression or E545K knock‐in mutation (hm or het) were subjected to subcellular fractionation (*n* = 3). GAPDH and lamin A/C are markers for cytosolic and nuclear fractions, respectively. (C) OVCAR8 cells with wild‐type *PIK3CA* overexpression or E545K mutation or DOV13 cells with wild‐type *PIK3CA* overexpression were co‐transfected with 8×GTIIC firefly luciferase and Renilla luciferase plasmids prior to dual‐luciferase reporter assay (*n* = 3). (D) RNA‐seq and GSEA analysis of hallmark gene sets representing YAP conserved signature using ranked gene expression changes in *PIK3CA* E545K homozygous (left) or heterozygous (right) knock‐in OVCAR8 cells compared with control cells (*n* = 3 per cell line). Normalized enrichment score (NES), *P*‐value, and FDR are shown. (E) Images of scratch assay using OVCAR8 and DOV13 cells stably expressing wild‐type YAP, or mutant S5A, S127A, or S127D were taken at 0 and 16 h after scratch was made. Quantification of percentage wound closure after 16 h is shown in the right bar charts (*n* = 5). Scale bar, 200 μm. (F, G) Active RAC1 pull‐down assay and western blotting analysis of (F) OVCAR8 or DOV13 cells stably expressing wild‐type YAP, S5A, S127A or S127D or (G) *PIK3CA* E545K knock‐in OVCAR8 cells, OVCAR8 or DOV13 cells with stable wild‐type *PIK3CA* overexpression (*n* = 3). All the graphs display the mean value ± SD. *P < 0.05; **P < 0.01; ***P < 0.001; ****P < 0.0001; ns, no significant difference compared with control or vector using one‐way ANOVA (A, E) or two‐way ANOVA (C) with Sidak's multiple comparison test. Numbers below blots are densitometric values normalized to that of ERK2 relative to the control.

Immunofluorescence assay was performed to examine the nucleocytoplasmic localization of YAP in *PIK3CA*‐overexpressing cells and the mutant cells. The data showed that cells with E545K or *PIK3CA* overexpression displayed stronger cytoplasmic staining of YAP than the control cells (Fig. 5A). The staining was quantified and presented as nuclear/cytoplasmic intensity ratios. The ratio was significantly lower in the *PIK3CA* mutant or overexpressing cells, indicating an accumulation of cytoplasmic YAP protein. Subcellular fractionation experiments revealed a 2‐ to 3‐fold reduction in nuclear YAP and 2.3‐fold elevation in cytoplasmic YAP (Fig. 5B). Accompanied with the decreased nuclear YAP level was a reduction in YAP transcriptional activity as revealed by luciferase reporter assay (Fig. 5C) and an enrichment of YAP‐associated gene signature based on RNA‐seq (Fig. 5D). Further analysis of the RNA‐seq data and real‐time PCR validation demonstrated that the mRNA levels of YAP transcriptional targets (such as *CCN1*, *CCN2*, and *BAX*) were reduced upon *PIK3CA* overexpression or E545K mutation in OVCAR8 and DOV13 cells (Fig. S4A–C), suggesting a diminished transcriptional activity of YAP.

This increase Ser127 phosphorylation and the localization changes of YAP as a result of the oncogenic *PIK3CA* aberrations led us to ask two questions: Whether YAP is oncogenic or tumor suppressive in the ovarian cancer cells and whether cytoplasmic YAP carries any function. First, to determine the functional role of YAP on tumor phenotypes, cell viability assay and migration assay were performed after YAP knockdown by siRNA (Fig. S5A). We observed a marked reduction in cell viability and migration capability (Fig. S5B,C), indicating an oncogenic role of YAP. Next, the phenotypic effects of Ser127 phosphorylation were studied by stably expressing a constitutively phosphorylated mutant (S127D) in OVCAR8 and DOV13 cells. Stable cell lines expressing wild‐type or phosphorylation‐defective mutant (S127A or S5A which has five phosphorylation sites mutated [22]) were also established and were included in parallel experiments for comparison. Expression levels of total YAP were similar across these stable cells, whereas Ser127 phosphorylation levels were lower in cells expressing S5A or S127A as expected (Fig. S6A). Concordant with the role of Ser127 in promoting cytoplasmic retention, the phospho‐mimicking S127D mutant caused decreases in nuclear YAP level and YAP transcriptional activity compared with wild‐type YAP or the two phospho‐dead mutants S5A and S127A (Fig. S6B,C).

Next, cell migration was assessed by wound healing assays. Strikingly, as shown in Fig. 5E, cells expressing S127D migrated more rapidly than GFP‐expressing control cells and in similar extent with wild‐type YAP or the phospho‐dead mutants. The pro‐migratory effect of YAP S127D could also be captured using time‐lapse imaging (Fig. S6D; Videos S1–, S3). S127D had no effect on cell viability because the viability of S127D‐expressing cells was comparable to that of control cells (Fig. S6E), suggesting that the effect of S127D on cell migration was independent of cell viability. Rho family GTPases such as RAC1 play key roles in cell migration events [33]. Consistent with an increased cell migration, the levels of active (GTP‐bound) RAC1 were higher upon expression of YAP S127D (Fig. 5F). The induction of RAC1 activity caused by S127D was stronger (2.7‐ to 3.9‐fold) compared to that caused by WT (1.8‐ to 2.1‐fold). Cells with the phospho‐dead mutant (S5A or S127A) demonstrated weaker RAC1 activation than those with WT YAP or S127D. We next determined RAC1 activity in *PIK3CA*‐aberrated cells. Remarkably, these cells also displayed higher RAC1 activity (2.0–3.5‐fold) (Fig. 5G).

### 3.6 N‐cadherin expression is increased upon *PIK3CA* aberrations

**Fig. 6:**
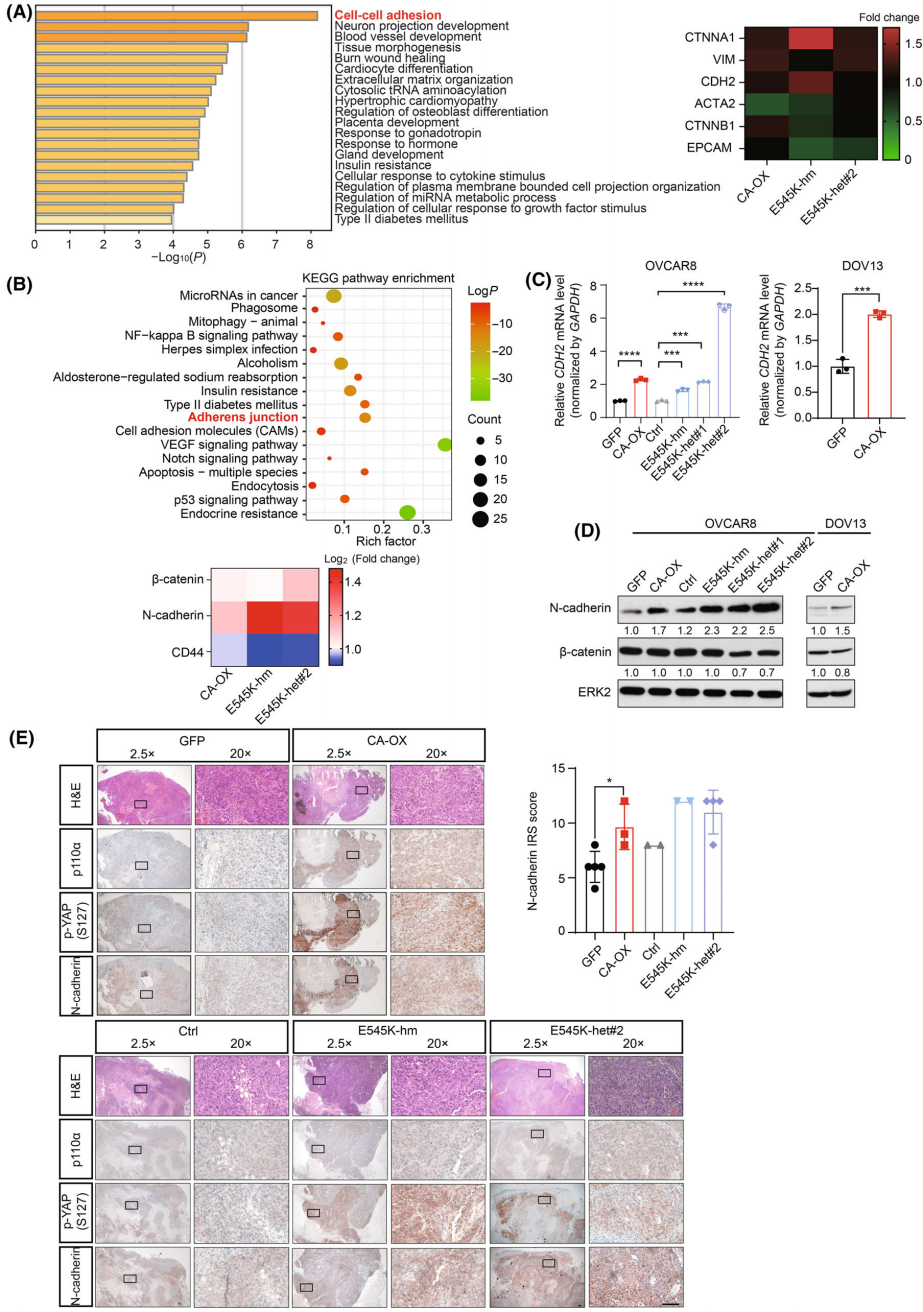
*PIK3CA* aberrations increased the mRNA and protein expression levels of N‐cadherin. (A) Left, gene ontology enrichment analysis was performed with the overlapping differentially expressed genes of wild‐type *PIK3CA*‐overexpressing (CA‐OX) OVCAR8 cells and homozygous (hm) or heterozygous (het) E545K knock‐in OVCAR8 cells. Fonts in red indicate annotation related to cell adhesion molecules. Right, the heatmap shows the mRNA levels of cell–cell adhesion molecules in wild‐type *PIK3CA*‐overexpressing OVCAR8 cells and E545K knock‐in OVCAR8 cells normalized to their corresponding control cells (*n* = 3 per cell line). (B) Top, the overlapping differentially expressed proteins between the wild‐type *PIK3CA* overexpressing OVCAR8 cells, E545K knock‐in OVCAR8 cells, and their corresponding control cells obtained from protein array data (*n* = 2 per cell line) were subjected to pathway enrichment analysis using Kyoto Encyclopedia of Genes and Genomes (KEGG) database and kobas software. Fonts in red indicate annotation related to cell adhesion molecules. The color of the circle indicates Log *P*‐value. The size of the circle is proportional to the number of enriched proteins. Bottom, the heatmap shows the levels of cell–cell adhesion proteins in wild‐type *PIK3CA*‐overexpressing OVCAR8 cells or E545K knock‐in OVCAR8 cells normalized to their corresponding control cells. (C) Total RNA of OVCAR8 or DOV13 cells with the indicated *PIK3CA* alterations was harvested for real‐time PCR analysis of *CDH2* levels (*n* = 3). *GAPDH* was used as an internal control. (D) OVCAR8 or DOV13 cells with the indicated *PIK3CA* alterations were harvested for western blotting (*n* = 3). ERK2 was a loading control. Numbers below blots are densitometric values normalized to that of ERK2 relative to the control. (E) Representative immunohistochemical images of xenograft tumors derived from *PIK3CA*‐overexpressing or E545K knock‐in cells stained with anti‐p110α antibody, anti‐p‐YAP (Ser127), or anti‐N‐cadherin antibody at 2.5× and 20×. Quantification of staining using immunoreactive score (IRS) is shown in the bar chart (*n* = 2–5). Scale bar, 100 μm. All the graphs display the mean value ± SD. *P < 0.05; ***P < 0.001; ****P < 0.0001 using one‐way ANOVA with Sidak's multiple comparison test.

We further mined the RNA‐seq and protein array data for the other molecular alterations driven by the *PIK3CA* aberrations. The DEGs (*n* = 172) or DEPs (*n* = 40) commonly found in *PIK3CA*‐overexpressing cells, homozygous, or heterozygous E545K knock‐in cells were subjected to GO enrichment analysis and KEGG pathway analysis. Intriguingly, we found that the intersecting DEGs and DEPs were significantly involved in cell adhesion (Fig. 6A,B). Among these, cell adhesion molecules were *CDH2* (N‐cadherin) and *CTNNB1* (β‐catenin) (Fig. 6A,B). We could confirm that the expression of *CDH2* (N‐cadherin) was consistently upregulated in the *PIK3CA*‐aberrated cells by real‐time PCR and western blot (Fig. 6C,D). As shown by IHC staining, N‐cadherin protein was strongly expressed in both *PIK3CA*‐overexpressing or E545K mutant xenograft tumors compared with control tumors (Fig. 6E). These *PIK3CA*‐aberrated tumors had concurrently high YAP Ser127 levels, which was consistent with the IHC data shown in Fig. 4B.

### 3.7 N‐cadherin antagonist inhibits the migration and metastasis of *PIK3CA*‐aberrated cells

**Fig. 7:**
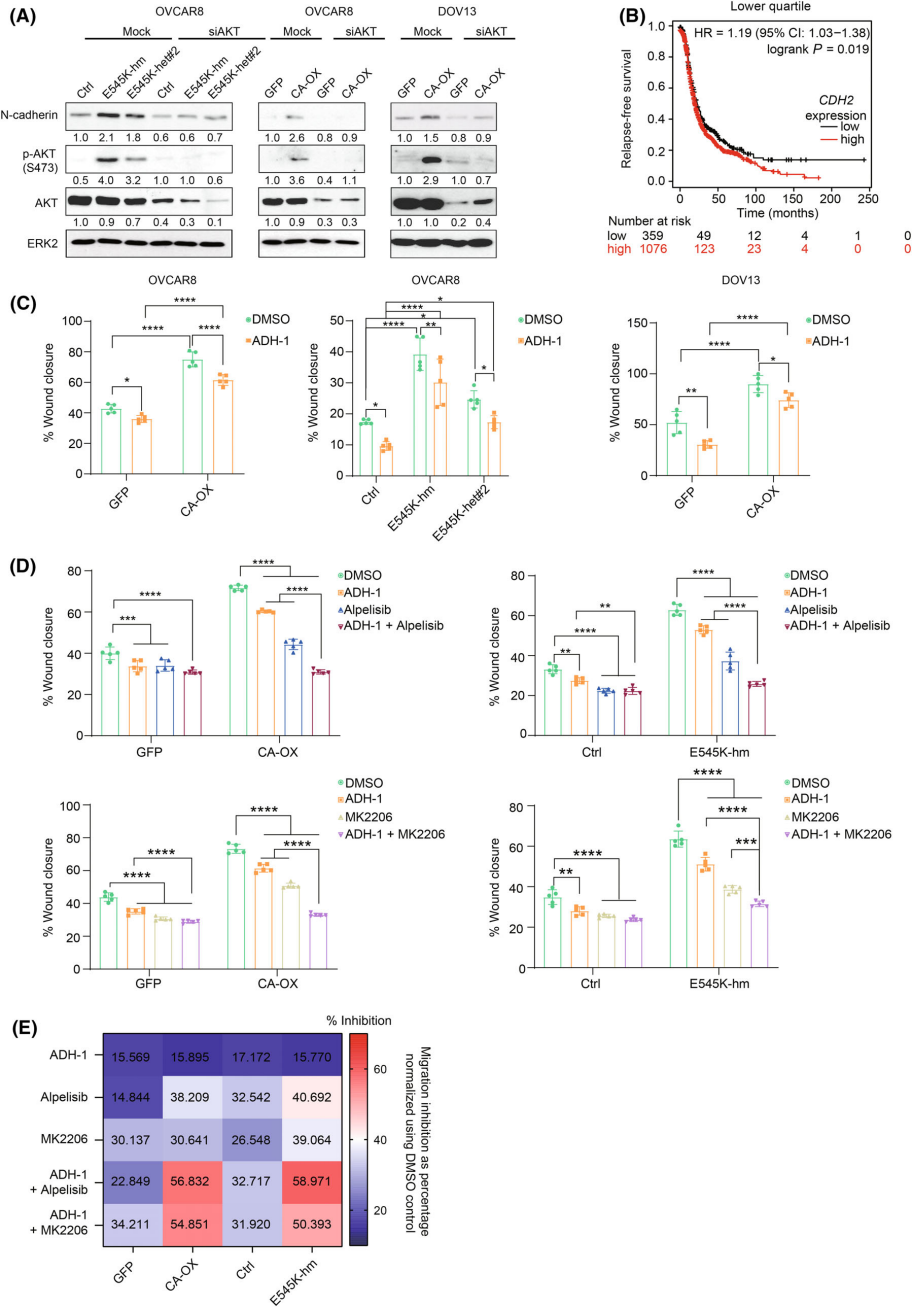
Suppressed migration of *PIK3CA*‐aberrated ovarian cancer cells upon inhibition of p110α, AKT, or N‐cadherin alone or in combination. (A) *PIK3CA* homozygous (hm) or heterozygous (het) E545K knock‐in OVCAR8 cells, OVCAR8 or DOV13 cells with stable wild‐type *PIK3CA* overexpression (CA‐OX) were transfected with *CDH2* siRNA for 72 h prior to western blotting (*n* = 3). ERK2 was used as loading control. Numbers below blots are densitometric values normalized to that of ERK2 relative to the control. (B) Relapse‐free survival of ovarian cancer patients stratified based on *CDH2* mRNA levels using lower quartile as cutoff. The analysis was generated by KM plotter using expression data obtained from Gene Expression Omnibus datasets. Hazard ratio (HR), 95% confidence interval (CI), and logrank *P*‐value are shown. (C) Quantification of percentage wound closure at 16 h after treatment with ADH‐1 (0.06 mg·mL^−1^) (*n* = 5). (D) *PIK3CA*‐overexpressing OVCAR8 cells, E545K knock‐in OVCAR8 cells and control cells were treated with (top) ADH‐1 at 0.06 mg·mL^−1^ or alpelisib at 10 μm alone or in combination or (bottom) ADH‐1 at 0.06 mg·mL^−1^ or MK2206 at 10 μm alone or in combination for 16 h (*n* = 5). (E) The heatmap shows the percentage of migration inhibition compared with DMSO based on the data in (D). All the plots show mean ± SD. *P < 0.05; **P < 0.01; ***P < 0.001; ****P < 0.0001 by two‐way ANOVA with Sidak's multiple comparison test.

Knockdown of AKT1/2/3 by siRNA markedly abrogated the induction of N‐cadherin expression in the *PIK3CA*‐aberrated cells, suggesting that the upregulation was a downstream effect of AKT (Fig. 7A). Our data also demonstrated that YAP Ser127 is not involved in regulating N‐cadherin expression. Wild‐type YAP, but not the phospho‐mimic mutant S127D, upregulated mRNA and protein levels of N‐cadherin (Fig. S7A,B). These results suggested that the increases in YAP Ser127 and N‐cadherin levels are likely two independent events downstream of AKT.

**Fig. 8:**
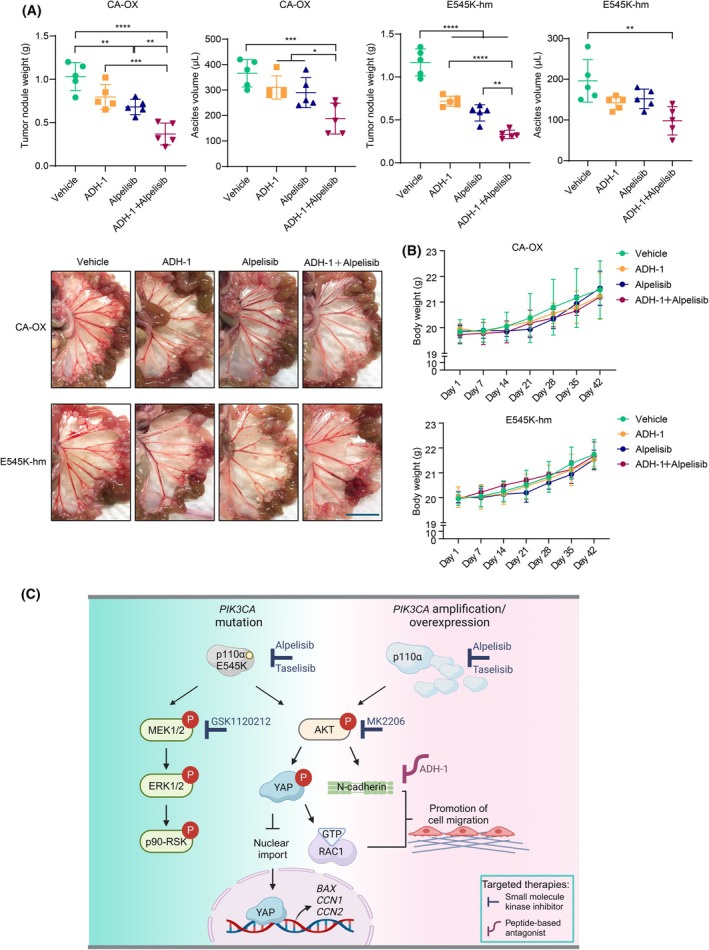
Inhibition of *in vivo* metastasis by alpelisib and ADH1. (A) Mice bearing *PIK3CA* homozygous (hm) E545K knock‐in or stable wild‐type *PIK3CA*‐overexpressing (CA‐OX) OVCAR8 cells (*n* = 5 per group) were treated with vehicle or ADH‐1 and/or alpelisib for 3 weeks. Tumor nodules and ascites collected after the treatment were measured. Pictures show representative mesentery with tumor nodules developed. Scale bar, 1 cm. Data shown are mean ± SD. *P < 0.05; **P < 0.01; ***P < 0.001; ****P < 0.0001 by one‐way ANOVA with Sidak's multiple comparison test. (B) Body weight analysis of mice in (A). Data shown are mean ± SD. (C) Proposed model of oncogenic signaling downstream of *PIK3CA* driver mutation or amplification. The signaling axis in the middle of this schematic diagram represents a common pathway induced by *PIK3CA* mutation or amplification. Both types of *PIK3CA* aberrations lead to AKT activation, which in turn phosphorylates YAP at Ser127. Phosphorylation of this residue causes (a) cytoplasmic retention of YAP and thereby reduced transcription of YAP target genes in the nucleus, such as the pro‐apoptotic *BAX*, and (b) enhanced RAC1 activity to promote cell migration. Another independent event downstream of AKT is an increased expression of N‐cadherin, which also contributes positively to cell migratory potential. Building on these mechanistic insights, we further showed that p110α, AKT, and N‐cadherin are therapeutic targets in *PIK3CA*‐aberrated serous ovarian cancer cells. *PIK3CA* mutation, but not amplification, induces the MAPK pathway. The figure was created with BioRender.com.

We then investigated the functional significance of N‐cadherin expression. N‐cadherin is a reported oncogene that promotes metastasis [34]. Aligning with the notion that metastasis is a strong indicator of patient prognosis, ovarian cancer patients with high *CDH2* mRNA levels were significantly associated with an unfavorable prognosis (Fig. 7B). Remarkably, inhibition of N‐cadherin by an antagonist (ADH‐1) led to reduction of cell migratory ability (Fig. 7C). However, cell viability was not decreased by either N‐cadherin (*CDH2*) siRNA or ADH‐1 (Fig. S8A,B). Given that vertical pathway inhibition (co‐targeting an upstream alteration and a downstream effector) may achieve stronger inhibitory outcome [35, 36] and our finding that AKT upregulates N‐cadherin expression, we sought to determine whether simultaneous targeting of N‐cadherin and p110α (alpelisib) or AKT (MK2206) is more effective in suppressing cell migration of *PIK3CA*‐aberrated ovarian cancer cells. Strikingly, dual inhibition of PI3K/AKT and N‐cadherin had stronger effect on blocking cell migration than either inhibition alone (Fig. 7D,E). *PIK3CA*‐aberrated cells were more sensitive to the combined inhibition compared with the control cells (Fig. 7D,E). While the combined treatment (ADH‐1 + alpelisib) caused 23–33% inhibition in control cells, *PIK3CA*‐overexpressing or E545K mutant cells showed 57–59% reduction in migration. The inhibitory effects of p110α‐specific inhibitor and AKT inhibitor in the *PIK3CA*‐aberrated cells were similar. To investigate the effect of p110α and N‐cadherin inhibitors on peritoneal metastasis *in vivo*, mice were i.p. injected with *PIK3CA*‐overexpressing or homozygous E545K cells and treated with alpelisib and/or ADH‐1. An inhibition of metastases formation upon treatment could be observed. Alpelisib single treatment resulted in reduced tumor metastasis to the mesentery and omentum, as evidenced by a decrease in tumor nodule weight (*P* < 0.01; Fig. 8A). ADH‐1 treatment also caused decreases in tumor nodule weight, with statistical significance only observed in E545K cells (*P* < 0.0001). The combination of alpelisib and ADH‐1 led to an additive suppression of tumor nodule formation, which was significantly greater than the effect observed with either inhibitor alone (*P* < 0.01). The efficacy of the drug combination was further demonstrated by the significant reduction in ascites volume compared to the vehicle control (*P* < 0.01; Fig. 8A). No toxicity was observed in mice receiving the drugs, either alone or in combination, as demonstrated by body weight (Fig. 8B).

## 4 Discussion

This study revealed the common and differential tumorigenic signaling pathways in the context of *PIK3CA* amplification and E545K mutation in serous ovarian cancer cells (Fig. 8C). Serous ovarian cancer is characterized by more frequent copy number alterations than small nucleotide mutations [13]. Therefore, *PIK3CA* amplification is predominant over *PIK3CA* mutation in the disease. Our findings together suggest *PIK3CA* amplification as an alternative to *PIK3CA* hotspot mutation (such as E545K) for serous ovarian tumorigenesis.

In light of the functional significance of p110α in driving cancers, p110α‐selective inhibitors have been developed to target this oncoprotein. Alpelisib, in combination with fulvestrant, is FDA‐approved for the treatment of hormone receptor‐positive, HER2‐negative, *PIK3CA*‐mutated, advanced, or metastatic breast cancer [37]. So far, *PIK3CA* mutation status has been indicated as the strongest predictive marker to identify patients who may derive benefit from alpelisib [38]. *In silico* analysis of Cancer Cell Line Encyclopedia data showed that while *PIK3CA* mutation is the top predictor for alpelisib sensitivity, *PIK3CA* amplification also positively associated with response to alpelisib [39]. Our findings presented here provide additional evidence supporting the potential of *PIK3CA* amplification as a biomarker for predicting response to p110α inhibitors. The clinical data of a phase 1b clinical trial investigating the combination of alpelisib and a PARP inhibitor olaparib in ovarian cancer patients have demonstrated encouraging efficacy [40]. Considering *PIK3CA* copy number status in patient selection may further increase the clinical benefit of this treatment approach.

It has been proposed that vertical inhibition, which involves targeting multiple points within the same pathway, can achieve greater therapeutic benefit than targeting a single point, as exemplified by the combination of BRAF and MEK inhibitors or AKT and mTOR inhibitors [35, 36]. The rationale is that vertical inhibition can more effectively block the signaling cascade and overcome potential negative feedback loop. Our results align with this proposal, as we observed that co‐inhibition of PI3K/AKT and its downstream target N‐cadherin achieves a greater therapeutic effect in suppressing cancer metastasis. Upregulation of N‐cadherin expression by the PI3K/AKT pathway has been documented [41, 42]. There is increasing evidence demonstrating that the overexpression of N‐cadherin is a common event in various tumor types and is associated with cancer aggressiveness and clinical stage [34]. N‐cadherin antagonists in the form of peptides or monoclonal antibodies have shown the ability to inhibit metastasis and tumor growth in preclinical cancer models [43]. ADH‐1 is an anti‐N‐cadherin peptide and the only N‐cadherin antagonist that has been evaluated in early clinical trials. Preliminary efficacy data showed that ADH‐1 was generally well‐tolerated, with some evidence of tumor response and disease stabilization in a subset of patients particularly those with gynecological cancers [44, 45]. Therefore, inhibition of N‐cadherin may represent a viable strategy for ovarian cancer treatment.

The nucleocytoplasmic distribution of YAP can be regulated by many pathways. The most classical regulator is the LATS1/2 kinases of the Hippo pathway which phosphorylate YAP at Ser127 [31, 46]. YAP has also been shown as a direct substrate of AKT, which phosphorylates YAP at Ser127 and promotes YAP localization to the cytoplasm [32]. Consistently, AKT‐dependent phosphorylation of YAP and enhanced YAP nuclear translocation was reported in HNSCC [47]. While most of the previous studies have focused on the activities of nuclear YAP as a transcriptional coactivator [48, 49, 50], there are accumulating reports on the potential oncogenic roles of cytoplasmic YAP. In colorectal cancer, the expression of cytoplasmic YAP was significantly higher when compared to the surrounding normal tissues and was significantly correlated with worse disease‐free survival [51]. On the contrary, nuclear YAP level did not correlate with prognosis [51]. In another colorectal cancer cohort, cytoplasmic YAP but not nuclear YAP level showed a positive correlation with tumor budding [52], which is indicative of metastasis and unfavorable prognosis. More examples include pediatric hepatocellular carcinoma which displayed prominent cytoplasmic YAP that was not seen in non‐neoplastic liver tissues and alveolar rhabdomyosarcoma which exhibited strong cytoplasmic YAP staining but weak nuclear staining [53, 54]. So far, two oncogenic effects caused by the cytoplasmic retention of YAP have been proposed. First, given the role of nuclear YAP in potentiating the transcriptional activity of p73, exclusion of YAP from the nucleus may compromise the transcription of p73 target genes which are mostly tumor suppressors such as the pro‐apoptotic *BAX* [32, 47]. Accordingly, sequestration of YAP in the cytoplasm reduced apoptosis of HNSCC [47]. We also observed reduction of *BAX* mRNA expression in the *PIK3CA*‐aberrated cells. The second oncogenic effect is caused by a nongenomic function of YAP. Cytoplasmic YAP but not nuclear YAP promotes endothelial cell migration through cell division cycle 42 (CDC42) [55]. The mechanism of CDC42/RAC1 activity induction by cytoplasmic YAP, for example, whether guanine‐nucleotide exchange factor or GTPase activating protein is involved, awaits further exploration.

## 5 Conclusions

In summary, this study demonstrates that the highly frequent *PIK3CA* amplification is akin to *PIK3CA* driver mutation in promoting tumorigenesis of serous ovarian cancer. We propose that simultaneous blockade of p110α/AKT and N‐cadherin could suppress cell viability (by p110α/AKT inhibitor) and cell migration (by p110α/AKT inhibitor and N‐cadherin antagonist) in *PIK3CA*‐aberrated ovarian cancer. This report provides the basis for a therapy to limit the dissemination of ovarian cancer.

## Conflict of interest

The authors declare no conflict of interest.

## Author contributions

LWTC conceived and designed the study. SZ, HIH, VCYM, and YZ planned and performed experiments. SZ, HIH, VCYM, YL, GZ, and LWTC performed data analysis, interpreted, and discussed the results. LWTC and SZ wrote the manuscript, with input from the other authors.

### Peer review

The peer review history for this article is available at https://www.webofscience.com/api/gateway/wos/peer‐review/10.1002/1878‐0261.13761.

## Supporting information


**Fig. S1.** High prevalence of *PIK3CA* amplification correlated with worse survival of serous ovarian cancer patients.
**Fig. S2.** Ovarian cancer cells with wild‐type *PIK3CA* overexpression or E545K knock‐in mutation acquired malignant phenotypes.
**Fig. S3.** Knockdown of LATS1/2 had no effect on the induced YAP Ser127 phosphorylation in the PIK3CA‐aberrated cells.
**Fig. S4.** The mRNA levels of YAP transcriptional targets were reduced in the presence of *PIK3CA* aberrations.
**Fig. S5.** Knockdown of YAP decreased viability and migration of OVCAR8 cells.
**Fig. S6.** YAP S127D‐expressing cells increased cell migration without any effect on cell viability.
**Fig. S7.** YAP S127D had no effect on the mRNA nor protein level of N‐cadherin.
**Fig. S8.** Inhibition of N‐cadherin had minimal effect on cell viability.

**Table S1.** Sequences of crRNA and single‐stranded donor template for *PIK3CA* E545K knock‐in.
**Table S2.** Sequences of siRNA used in this study.
**Table S3.** List of inhibitors used in this study.
**Table S4.** List of antibodies used in this study.
**Table S5.** Primer sequences for real‐time PCR.

**Video S1.** Time lapse imaging of migration of cells expressing GFP control.

**Video S2.** Time lapse imaging of migration of cells expressing wild‐type YAP.

**Video S3.** Time lapse imaging of migration of cells expressing YAP S127D.

## Data Availability

Data from this study are available from the corresponding author upon reasonable request.
