# Supplementary material for: Vertical inhibition of p110α/AKT and N‐cadherin enhances treatment efficacy in PIK3CA‐aberrated ovarian cancer cells
Source: Mol Oncol. 2024 Nov 14;19(4):1132–54. doi: 10.1002/1878-0261.13761 (PMC11977650; doi:10.1002/1878-0261.13761)
Supplement: Supplementary file 1 — Fig. S1. High prevalence of PIK3CA amplification correlated with worse survival of serous ovarian cancer patients. Fig. S2. Ovarian cancer cells with wild‐type PIK3CA overexpression or E545K knock‐in mutation acquired malignant phenotypes. Fig. S3. Knockdown of LATS1/2 had no effect on the induced YAP Ser127 phosphorylation in the PIK3CA‐aberrated cells. Fig. S4. The mRNA levels of YAP transcriptional targets were reduced in the presence of PIK3CA aberrations. Fig. S5. Knockdown of YAP decreased viability and migration of OVCAR8 cells. Fig. S6. YAP S127D‐expressing cells increased cell migration without any effect on cell viability. Fig. S7. YAP S127D had no effect on the mRNA nor protein level of N‐cadherin. Fig. S8. Inhibition of N‐cadherin had minimal effect on cell viability. [file MOL2-19-1132-s002.pdf]

Supplementary Figure 1

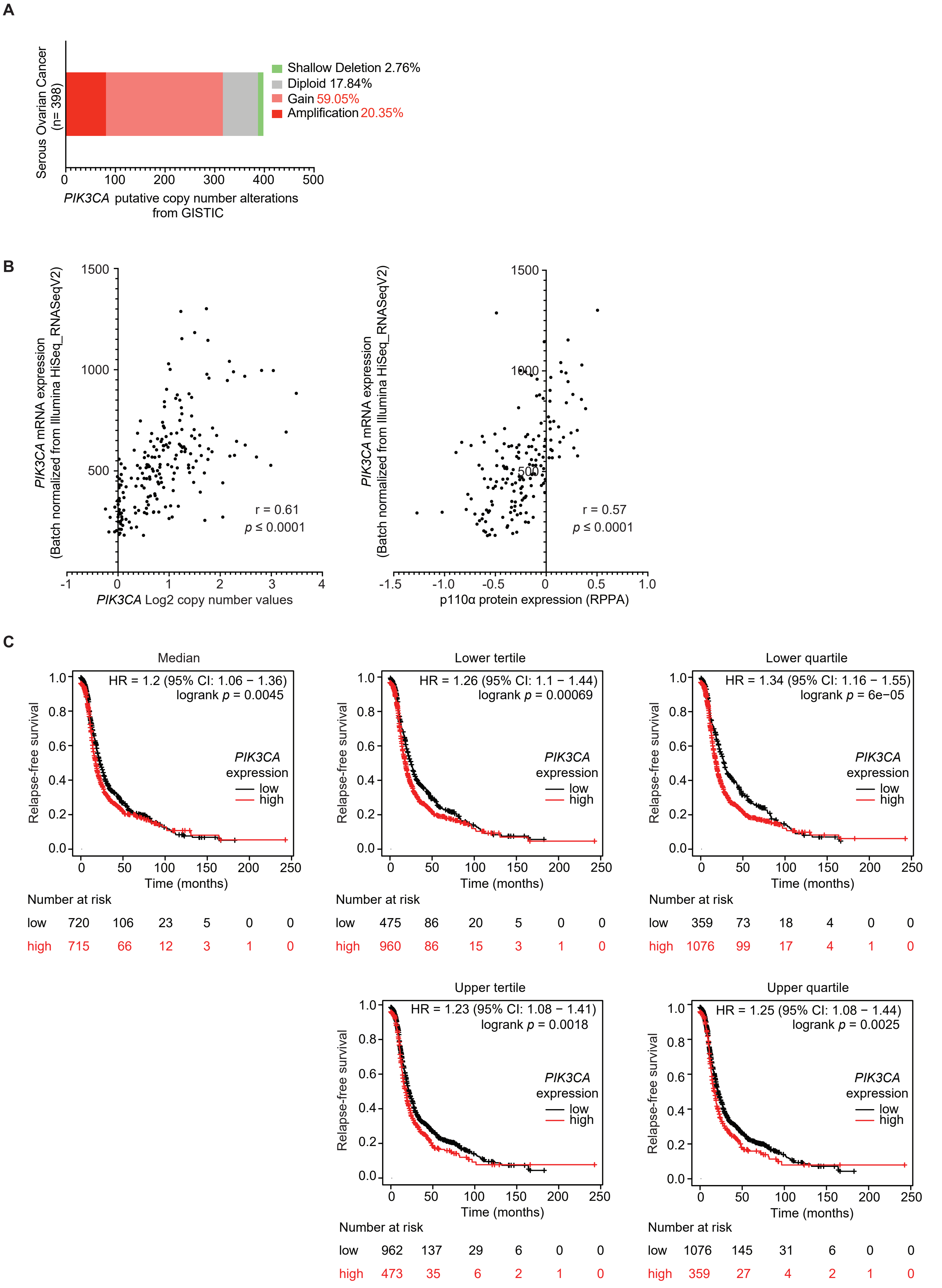

Supplementary Figure 1. High prevalence of *PIK3CA* amplification correlated with worse survival of serous ovarian cancer patients

(A) Frequency of *PIK3CA* copy number variations in TCGA serous ovarian cancer patients (n = 398). (B) Correlation between *PIK3CA* mRNA levels and *PIK3CA* copy numbers or p110α protein levels in TCGA serous ovarian cancer samples. Pearson correlation (r) and p-values are shown. (C) Relapse-free survival of ovarian cancer patients stratified based on *PIK3CA* mRNA levels using median, lower tertile, lower quartile, upper tertile or upper quartile as cutoff. The analysis was generated by KM plotter using expression data obtained from Gene Expression Omnibus datasets. Hazard ratio (HR), 95% confidence interval (CI), and logrank p-values are shown.

Supplementary Figure 2

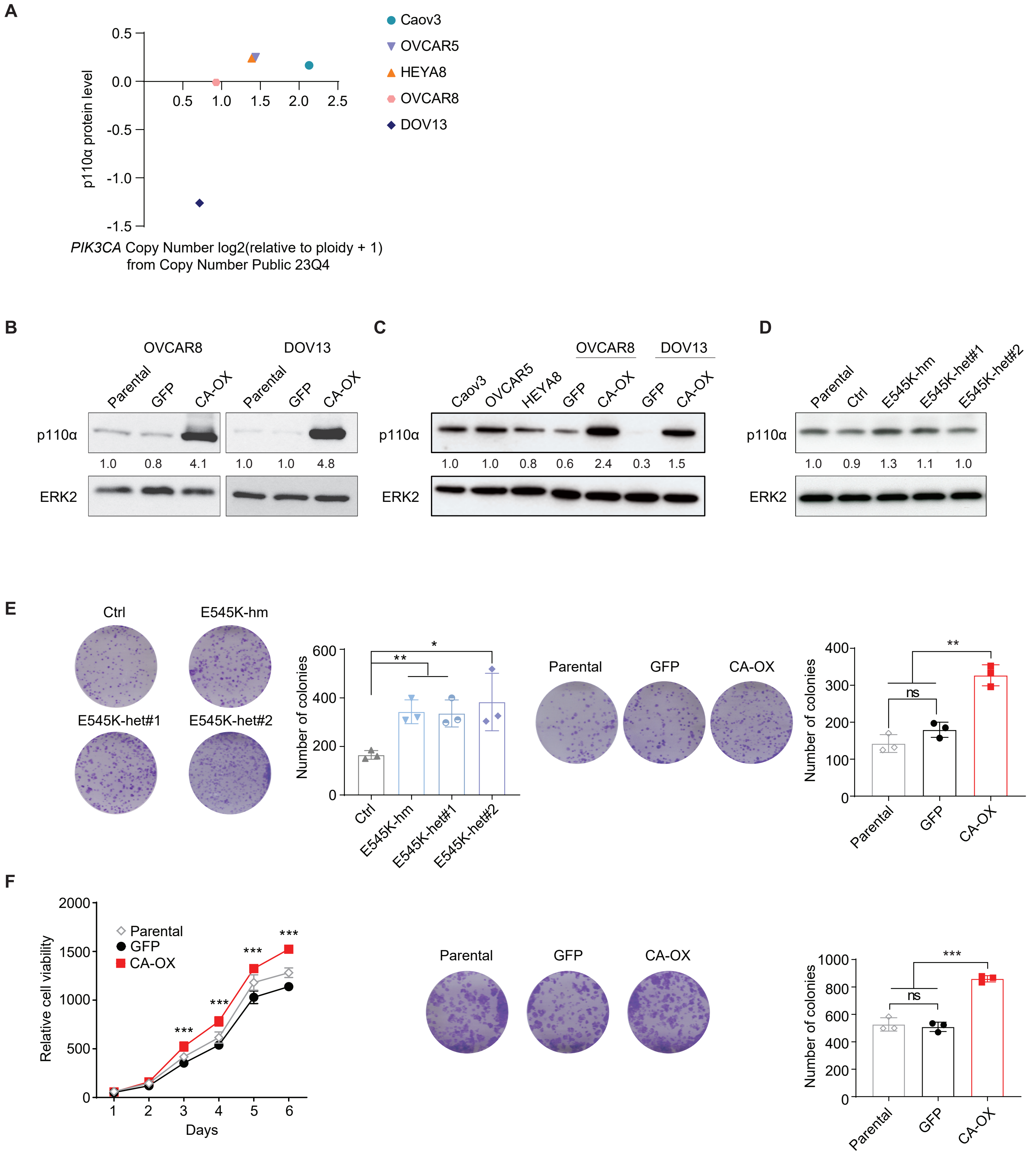

Supplementary Figure 2. Ovarian cancer cells with wild-type *PIK3CA* overexpression or E545K knock-in mutation acquired malignant phenotypes

(A) *PIK3CA* copy numbers from Copy Number Public 23Q4 and expression levels of p110α of the ovarian cancer cells were downloaded from Cancer Cell Line Encyclopedia (CCLE). (B-C) The protein levels of p110α in parental serous ovarian cancer cells or cells with stable overexpression (OX) of wild-type *PIK3CA* or empty vector (GFP) were verified by western blotting. (D) Lysates of parental OVCAR8 or OVCAR8 with CRISPR knock-in *PIK3CA* E545K mutant cells or unedited control cells were subjected to western blotting. ERK2 was loading control. Numbers below blots are densitometric values normalized to that of ERK2 relative to the control. (E) Left, *PIK3CA* E545K knock-in OVCAR8 cells and unedited control cells, or right, wild-type *PIK3CA*-overexpressing OVCAR8 cells or GFP control were seeded into 6-well plates and allowed to grow for 8 days (n=3). Representative images of colonies stained with crystal violet are shown. (F) Left, viability of DOV13 cells with stably overexpressing wild-type *PIK3CA* or GFP vector was measured at the indicated time points (n=3). Middle, wild-type *PIK3CA*-overexpressing DOV13 cells were allowed to grow for 8 days. Representative images of colonies are shown. Right, mean colony numbers ± SD (n=3). *p < 0.05; **p < 0.01; ***p < 0.001; ns, no significant difference using one-way (E, F) or two-way ANOVA (F, left) with Sidak's multiple comparison test.

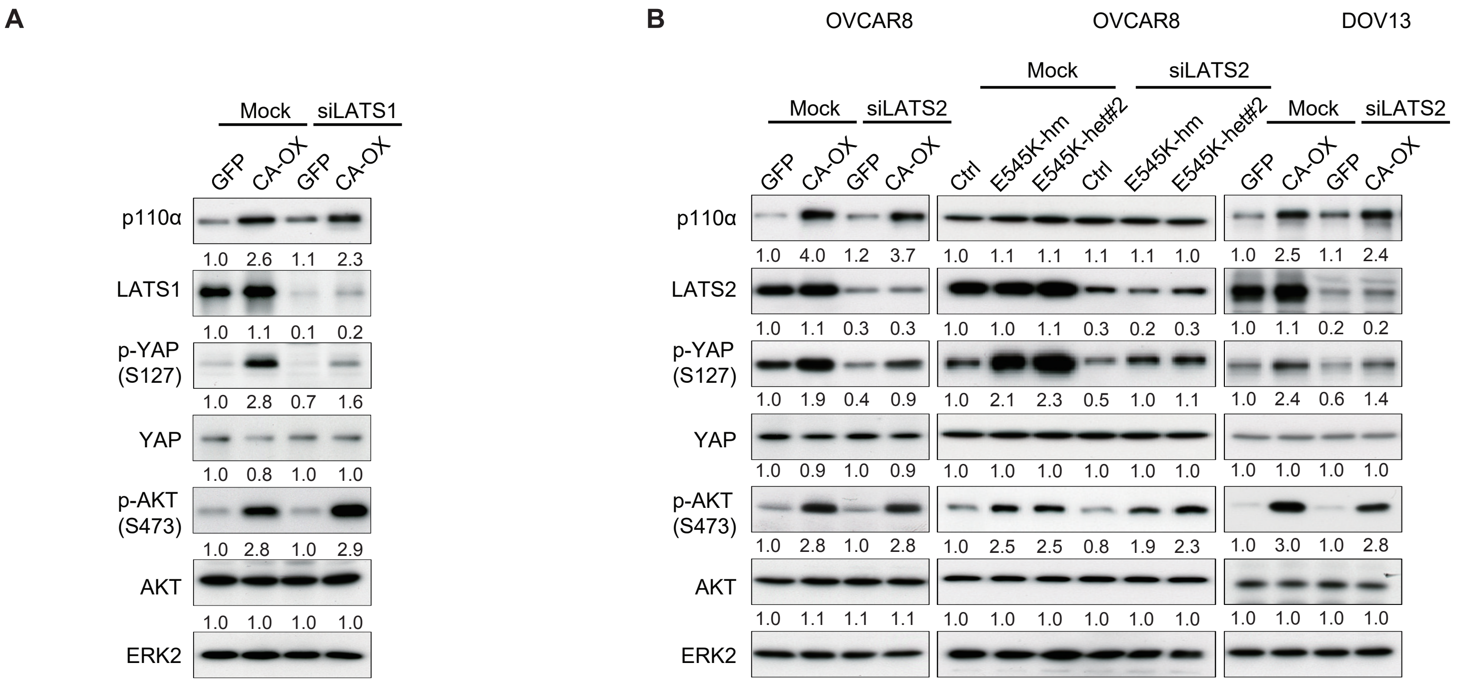

Supplementary Figure 3. Knockdown of LATS1/2 had no effect on the induced YAP Ser127 phosphorylation in the *PIK3CA*-aberrated cells

(A) DOV13 cells stably expressing wild-type *PIK3CA* or GFP vector were transfected with *LATS1* siRNA for 72 hr before western blotting (n=3). (B) Lysates of OVCAR8 cells stably expressing wild-type *PIK3CA* or E545K knock-in mutation or DOV13 cells stably expressing wild-type *PIK3CA* transfected with *LATS2* siRNA for 72 hr were subjected to western blotting (n=3). ERK2 was used as loading control. Numbers below blots are densitometric values normalized to that of ERK2 relative to the control.

Supplementary Figure 4

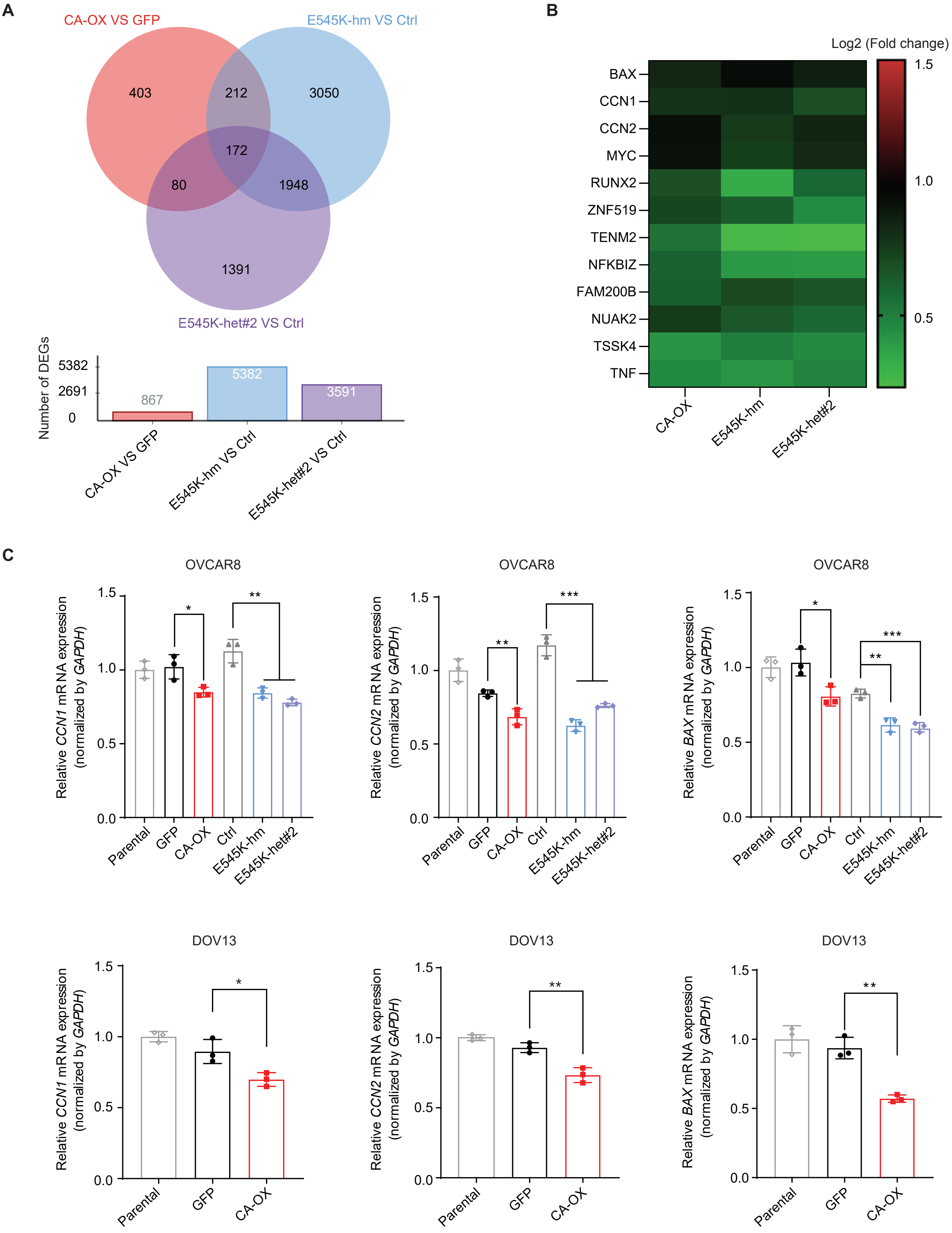

Supplementary Figure 4. The mRNA levels of YAP transcriptional targets were reduced in the presence of *PIK3CA* aberrations

(A) Venn diagram shows the overlap of DEGs between the wild-type *PIK3CA* overexpressing OVCAR8 cells compared with GFP vector cells and *PIK3CA* E545K knock-in OVCAR8 cells compared with corresponding control cells (n=3 per cell line). (B) The heatmap shows mRNA levels of YAP transcriptional targets in *PIK3CA*-overexpressing OVCAR8 cells, *PIK3CA* E545K knock-in cells normalized to their corresponding controls. (C) Total RNA of OVCAR8 and DOV13 cells with the indicated *PIK3CA* alterations was harvested for real-time PCR (n=3). *GAPDH* was internal control. The plots show mean ± SD. * $p < 0.05$; ** $p < 0.01$; *** $p < 0.001$ compared with control or vector using one-way ANOVA with Sidak's multiple comparison test. .

Supplementary Figure 5

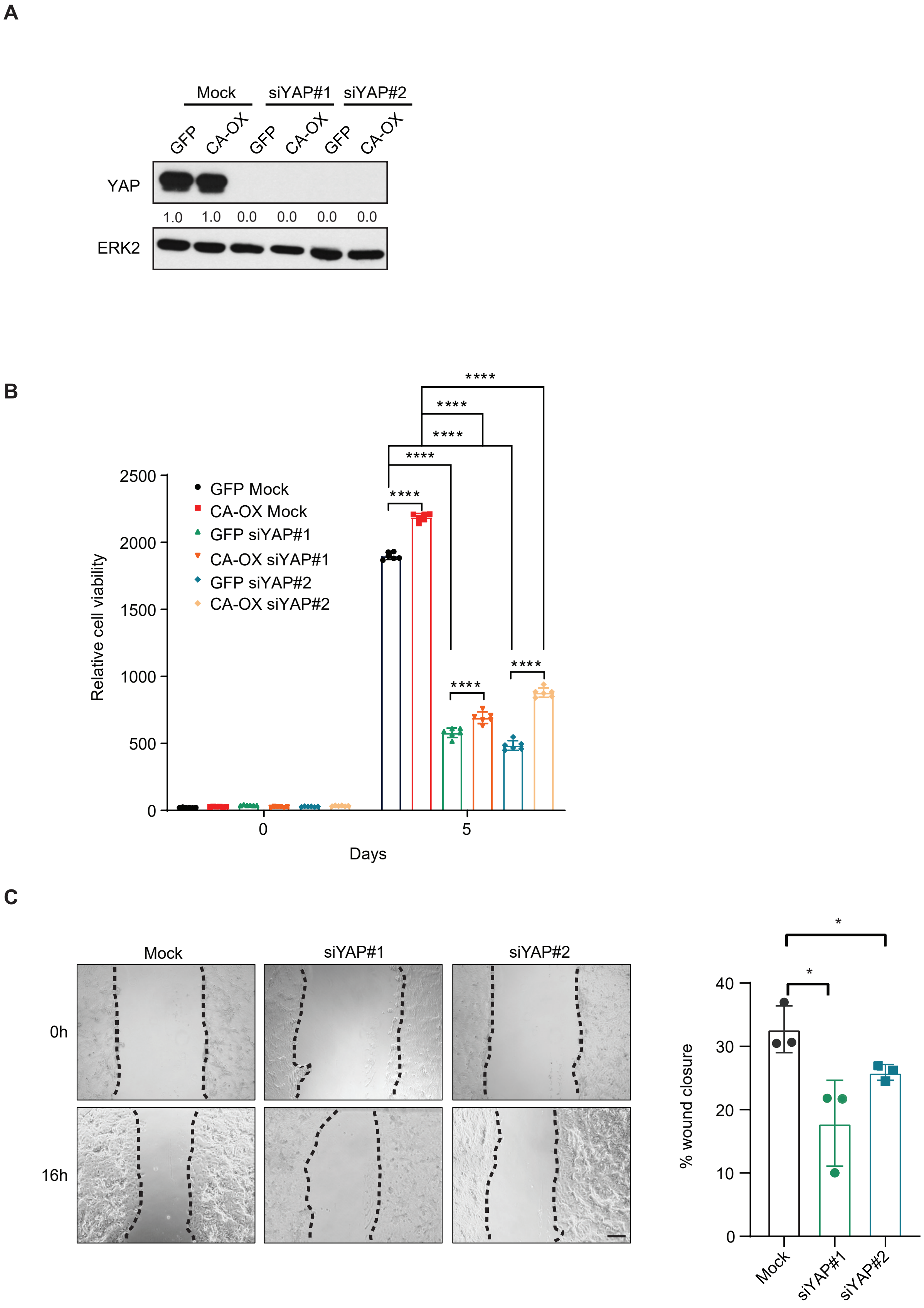

Supplementary Figure 5. Knockdown of *YAP* decreased viability and migration of OVCAR8 cells

(A) Lysates of OVCAR8 cells stably expressing wild-type *PIK3CA* transfected with *YAP1* siRNA for 72 hr were subjected to western blotting. ERK2 was used as loading control. Numbers below blots are densitometric values normalized to that of ERK2 relative to the control. (B) Viability of OVCAR8 cells stably expressing wild-type *PIK3CA* transfected with *YAP1* siRNA for 72 hr was measured over 7 days (n=5). Day 0 was the day of cell seeding. (C) Scratch assay of OVCAR8 cells transfected with *YAP1* siRNA for 24 hr. Images were taken at 0 hr and 16 hr after scratch was made. Scale bar, 200 μm. The graph shows mean of percentage wound closure ± SD from 3 different fields. *$p < 0.05$; ****$p < 0.0001$ compared with control or vector using one-way (C) or two-way (B) ANOVA with Sidak's multiple comparison test.

Supplementary Figure 6

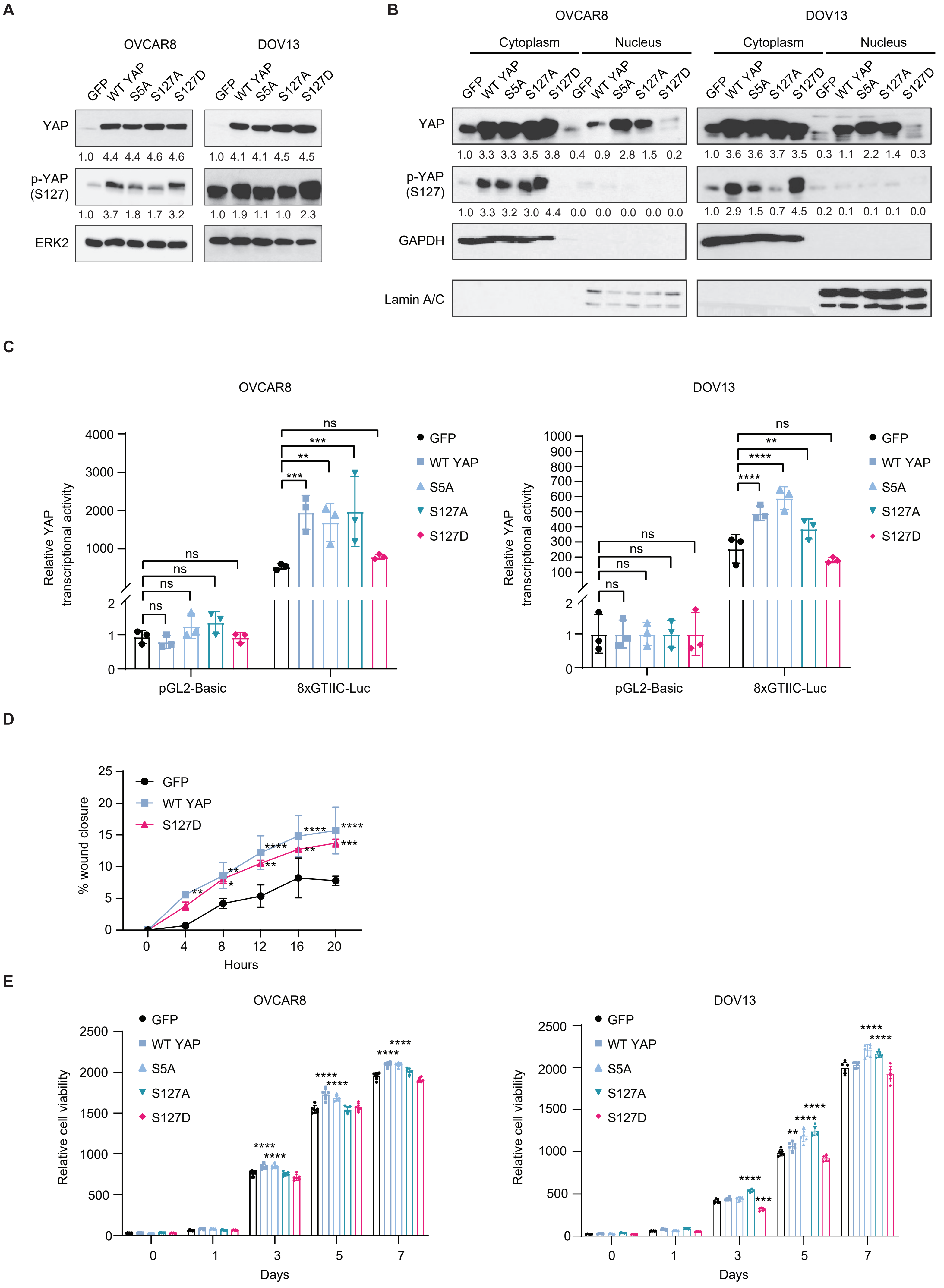

Supplementary Figure 6. YAP S127D-expressing cells increased cell migration without any effect on cell viability

(A) Lysates of OVCAR8 or DOV13 cells stably expressing wild-type YAP or mutant S5A, S127A or S127D were subjected to western blotting. ERK2 was used as loading control. Numbers below blots are densitometric values normalized to that of ERK2 relative to the control. (B) Lysates of the cells were harvested for subcellular fractionation before western blotting. GAPDH and lamin A/C are markers for the cytosolic and nuclear fractions, respectively. (C) The cells were co-transfected with pRL-TK Renilla luciferase plasmids and 8xGTIIC firefly luciferase or pGL2-Basic (vector backbone without 8xGTIIC-luciferase) plasmids (n=3). Dual-luciferase reporter assay was performed, and the normalized firefly/Renilla luciferase activities are presented. (D) Quantification of percentage of wound closure at indicated time points after scratch made at 0 hr was detected by IN Cell Analyzer 6500HS System (n=3). (E) Viability of OVCAR8 or DOV13 cells stably expressing wild-type YAP or mutant S5A, S127A and S127D was measured over 7 days (n=6). Day 0 was the day of cell seeding. Data show mean ± SD. * $p < 0.05$; ** $p < 0.01$; *** $p < 0.001$; **** $p < 0.0001$; ns, no significant difference by two-way ANOVA with Sidak's multiple comparison test.

Supplementary Figure 7

A

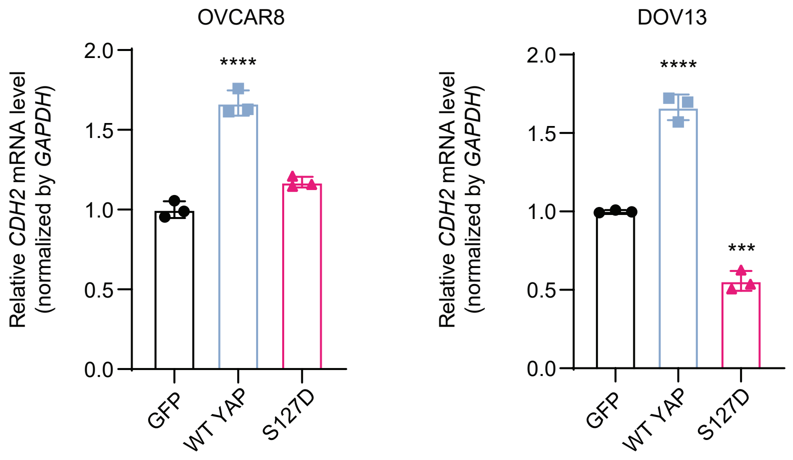

B

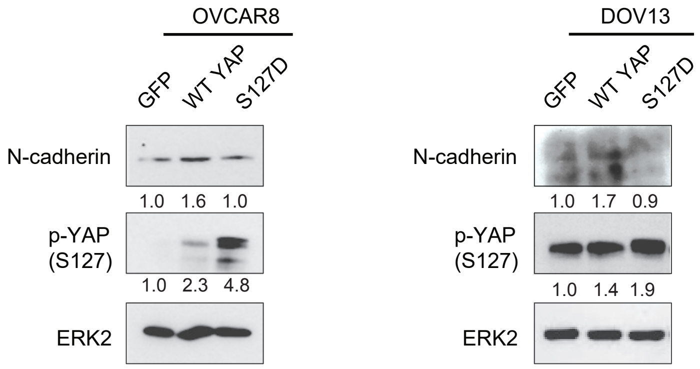

Supplementary Figure 7. YAP S127D had no effect on the mRNA nor protein level of N-cadherin

(A) Total RNA of OVCAR8 or DOV13 cells stably expressing wild-type YAP or mutant S127D was harvested for real-time PCR (n=3). GAPDH was internal control. (B) Lysates of OVCAR8 or DOV13 cells stably expressing wild-type YAP or mutant S127D were subjected to western blotting (n=3). ERK2 was used as loading control. Numbers below blots are densitometric values normalized to that of ERK2 relative to the control. Data are presented as mean ± SD. ***$p < 0.001$; ****$p < 0.0001$ using one-way ANOVA with Sidak's multiple comparison test.

Supplementary Figure 8

A

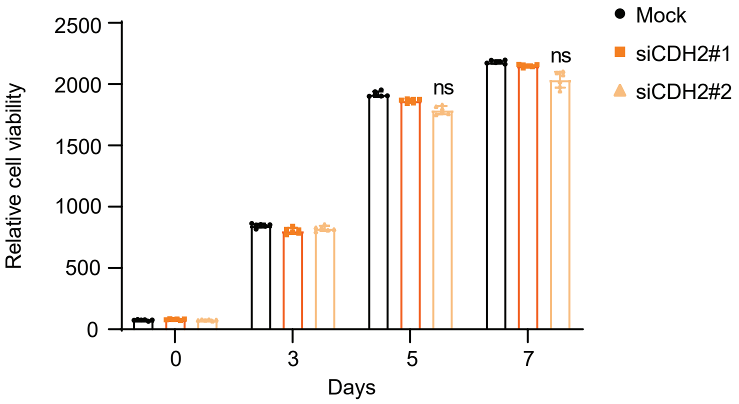

B

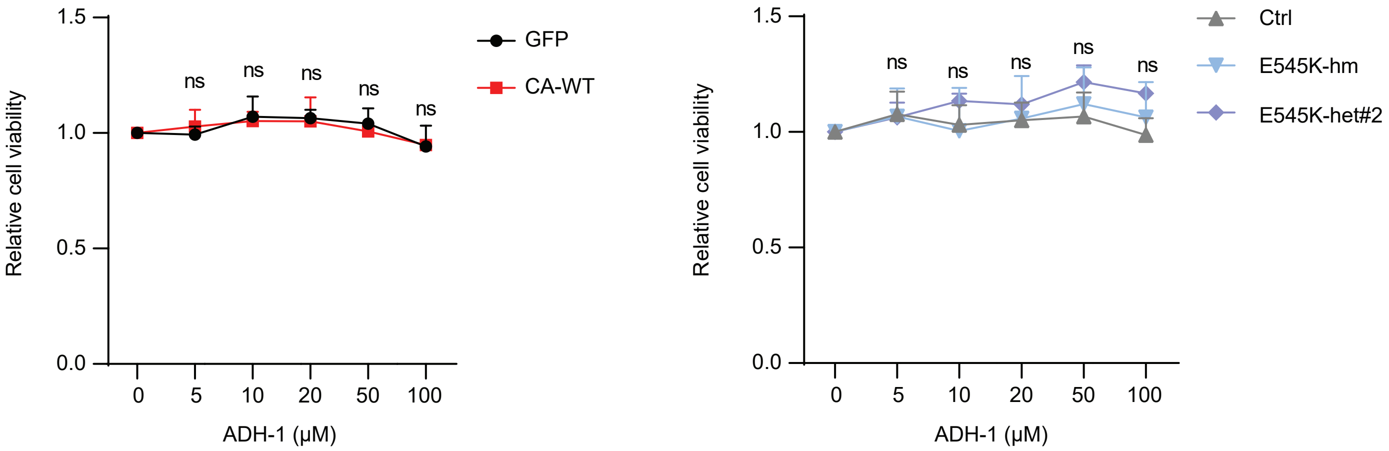

Supplementary Figure 8. Inhibition of N-cadherin had minimal effect on cell viability
(A) OVCAR8 cells were transfected with *CDH2* siRNA for 24 hr before being seeded into 96-well plates (n=5). Cell viability was measured over 7 days. Day 0 was the day of cell seeding. (B) OVACR8 cells stably expressing wild-type *PIK3CA* or with E545K knock-in mutation were treated with serial concentrations of ADH-1 for 72 hr. The plots show mean ± SD of triplicates. ns, no significant difference compared with vector or control by two-way ANOVA with Sidak’s multiple comparison test.
