# Supplementary material for: Vertical inhibition of p110α/AKT and N‐cadherin enhances treatment efficacy in PIK3CA‐aberrated ovarian cancer cells
Source: Mol Oncol. 2024 Nov 14;19(4):1132–54. doi: 10.1002/1878-0261.13761 (PMC11977650; doi:10.1002/1878-0261.13761)
Supplement: Supplementary file 2 — Table S1. Sequences of crRNA and single‐stranded donor template for PIK3CA E545K knock‐in. Table S2. Sequences of siRNA used in this study. Table S3. List of inhibitors used in this study. Table S4. List of antibodies used in this study. Table S5. Primer sequences for real‐time PCR. [file MOL2-19-1132-s004.docx]

**Supplementary Table 1.** Sequences of crRNA and single-stranded donor template for *PIK3CA* E545K knock-in

|  | **Sequence** |
| --- | --- |
| crRNA | TCTCTCTGAAATCACTGAGC |
| Single-stranded  donor template | AGAACAGCTCAAAGCAATTTCTACGCGAGATCCT  CTCTCTGAAATAACGAAGCAAGAGAAAGATTTTC  TATGGAGTCACAGGTAAGTGCTAAAATGGAGA |

**Supplementary Table 2.** Sequences of siRNA used in this study

| **Gene** | **Sequence** | |
| --- | --- | --- |
| *PIK3CA* | #1 | GUGGUAAAGUUCCCAGAUA |
|  | #2 | AAUGUAAAUAUUCGAGACAUUGAUA |
| *AKT1* | | CAAGGGCACUUUCGGCAAG |
| *AKT2* | | ACACAAGGUACUUCGAUGA |
| *AKT3* | | GCACACACUCUAACUGAAA |
| *LATS1* | | GGUGAAGUCUGUCUAGCAA |
| *LATS2* | | GCACGCAUUUUACGAAUUC |
| *YAP1* | #1 | GCUGCCACCAAGCUAGAUAAAGAAA |
|  | #2 | GGUCAGAGAUACUUCUUAAAUCACA |
| *CDH2* | #1 | CGAGAAUCACCAAAUGUG |
|  | #2 | AGGUCUGAUAGAGAUAAA |

**Supplementary Table 3.** List of inhibitors used in this study

| **Inhibitor name** | **Targets** | **Supplier** | **Catalog number** |
| --- | --- | --- | --- |
| Alpelisib  (BYL719) | PI3Kα inhibitor | Selleckchem  (Houston, TX) | S2814 |
| Taselisib  (GDC-0032) | PI3K inhibitor | MedChemExpress  (Monmouth Junction, NJ) | HY-13898 |
| PIK90 | PI3K inhibitor | Selleckchem | S1187 |
| MK-2206 | AKT inhibitor | Selleckchem | S1078 |
| Trametinib  (GSK1120212) | MEK1/2 inhibitor | Selleckchem | S2673 |
| ADH-1 | N-cadherin antagonist | MedChemExpress | HY-13541 |

**Supplementary Table 4.** List of antibodies used in this study

| **Protein** | **Supplier** | **Catalog**  **number** | **WB^#^**  **dilution** | **IF^#^**  **dilution** | **IHC^#^**  **dilution** |
| --- | --- | --- | --- | --- | --- |
| p110α | Cell Signaling Technology  (Danvers, MA) | 4249 | 1/1000 |  | 1/200 |
| AKT pS473 | Cell Signaling Technology | 9271 | 1/1000 |  |  |
| AKT pS308 | Santa Cruz Biotechnology  (Dallas, TX) | sc-271966 | 1/1000 |  |  |
| AKT | Cell Signaling Technology | 4691 | 1/3000 |  |  |
| PDK1 pS241 | Cell Signaling Technology | 3061 | 1/1000 |  |  |
| PDK1 | Cell Signaling Technology | 3062 | 1/1000 |  |  |
| PRAS40 pT246 | Cell Signaling Technology | 2997 | 1/1000 |  |  |
| PRAS40 | Cell Signaling Technology | 2610 | 1/1000 |  |  |
| S6 pS235/236 | Cell Signaling Technology | 2211 | 1/1000 |  |  |
| S6 | Cell Signaling Technology | 2317 | 1/1000 |  |  |
| ERK1/2  pT202/Y204 | Cell Signaling Technology | 9101 | 1/2500 |  |  |
| ERK1/2 | Cell Signaling Technology | 9102 | 1/5000 |  |  |
| p90RSK  pT359/S363 | Cell Signaling Technology | 9344 | 1/1000 |  |  |
| p90RSK | Cell Signaling Technology | 9333 | 1/1000 |  |  |
| MEK1/2  pS217/221 | Cell Signaling Technology | 9154 | 1/1000 |  |  |
| MEK1 | Santa Cruz Biotechnology | sc-6250 | 1/250 |  |  |
| LATS1 pS909 | Cell Signaling Technology | 9157 | 1/1000 |  |  |
| LATS1 | Cell Signaling Technology | 3477 | 1/1000 |  |  |
| LATS2 | Cell Signaling Technology | 5888 | 1/1000 |  |  |
| YAP pS127 | Cell Signaling Technology | 4911 | 1/1000 |  | 1/200 |
| YAP | Santa Cruz Biotechnology | sc-101199 |  | 1/200 |  |
| YAP | Cell Signaling Technology | 4912 | 1/1000 |  |  |
| N-cadherin | Cell Signaling Technology | 13116 | 1/1000 |  | 1/100 |
| β-catenin | Santa Cruz Biotechnology | sc-7963 | 1/1000 |  |  |
| PTEN | Cell Signaling Technology | 5384 | 1/1000 |  |  |

| Rac1 | Millipore  (Burlington, MA) | 05-389 | 1/1000 |  |  |
| --- | --- | --- | --- | --- | --- |
| ERK2 | Santa Cruz Biotechnology | sc-154 | 1/8000 |  |  |
| Lamin A/C | Cell Signaling Technology | 2032 | 1/1000 |  |  |
| GAPDH | GenScript  (Piscataway, NJ) | A00192 | 1/20000 |  |  |
| HRP-linked anti-  rabbit IgG  secondary antibody | GE Healthcare Life Sciences  (Chicago, IL) | NA931 | 1/5000 |  |  |
| HRP-linked anti-  mouse IgG  secondary antibody | GE Healthcare Life Sciences | NA934 | 1/5000 |  |  |

**^#^** WB, western blotting; IF, immunofluorescence; IHC, immunohistochemistry

**Supplementary Table 5.** Primer sequences for real-time PCR

| **Gene** | **Sequences** | |
| --- | --- | --- |
| *CCN1* | Forward | GGAAAAGGCAGCTCACTGAAGC |
|  | Reverse | GGAGATACCAGTTCCACAGGTC |
| *CCN2* | Forward | CTTGCGAAGCTGACCTGGAAGA |
|  | Reverse | CCGTCGGTACATACTCCACAGA |
| *BAX* | Forward | TCAGGATGCGTCCACCAAGAAG |
|  | Reverse | TGTGTCCACGGCGGCAATCATC |
| *CDH2* | Forward | CCTCCAGAGTTTACTGCCATGAC |
|  | Reverse | GTAGGATCTCCGCCACTGATTC |
| *GAPDH* | Forward | TCCATGACAACTTTGGTATCGTG |
|  | Reverse | ACAGTCTTCTGGGTGGCAGTG |
